# Analysis of Nucleotide Sequence of Tax, miRNA and LTR of Bovine Leukemia Virus in Cattle with Different Levels of Persistent Lymphocytosis in Russia

**DOI:** 10.3390/pathogens10020246

**Published:** 2021-02-20

**Authors:** Aneta Pluta, Natalia V. Blazhko, Charity Ngirande, Thomas Joris, Luc Willems, Jacek Kuźmak

**Affiliations:** 1 Department of Biochemistry, National Veterinary Research Institute, 24-100 Puławy, Poland; jkuzmak@piwet.pulawy.pl; 2 Research Center Innovations, 630039 Novosibirsk, Russia; 9137234044@mail.ru; 3 Laboratory of Enzyme Analysis and DNA Technologies, Novosibirsk State Agrarian University, St. Dobrolyubova, 630039 Novosibirsk, Russia; charityngirande@gmail.com; 4 Molecular and Cellular Epigenetics (Interdisciplinary Cluster for Applied Genoproteomics, GIGA) and Molecular Biology (TERRA), University of Liège (ULiège), 4000 Liege, Belgium; thom.joris@gmail.com (T.J.); luc.willems@uliege.be (L.W.)

**Keywords:** bovine leukemia virus (BLV), long terminal repeat (LTR), Tax protein, miRNA, persistent lymphocytosis, proviral load, sequence variants, primary isolates

## Abstract

Bovine Leukemia Virus (BLV) is the etiological agent of enzootic bovine leucosis (EBL), a lymphoproliferative disease of the bovine species. In BLV-infected cells, the long terminal repeat (LTR), the viral Tax protein and viral miRNAs promote viral and cell proliferation as well as tumorigenesis. Although their respective roles are decisive in BLV biology, little is known about the genetic sequence variation of these parts of the BLV genome and their impact on disease outcome. Therefore, the objective of this study was to assess the relationship between disease progression and sequence variation of the BLV Tax, miRNA and LTR regions in infected animals displaying either low or high levels of persistent lymphocytosis (PL). A statistically significant association was observed between the A(+187)C polymorphism in the downstream activator sequence (DAS) region in LTR (*p*-value = 0.00737) and high lymphocytosis. Our study also showed that the mutation A(−4)G in the CAP site occurred in 70% of isolates with low PL and was not found in the high PL group. Conversely, the mutations G(−133)A/C in CRE2 (46.7%), C(+160)T in DAS (30%) and A(310)del in BLV-mir-B4-5p, A(357)G in BLV-mir-B4-3p, A(462)G in BLV-mir-B5-5p, and GA(497–498)AG in BLV-mir-B5-3p (26.5%) were often seen in isolates with high PL and did not occur in the low PL group. In conclusion, we found several significant polymorphisms among BLV genomic sequences in Russia that would explain a progression towards higher or lower lymphoproliferation. The data presented in this article enabled the classification between two different genotypes; however, clear association between genotypes and the PL development was not found.

## 1. Introduction

Bovine leukaemia virus (BLV), which belongs to the *Retroviridae* family and *Deltaretrovirus* genus, is the etiologic agent of enzootic bovine leucosis (EBL). BLV infection is found throughout the world, but its prevalence varies in different geographic locations, remaining the highest in the Americas and Asia. In the Russian Federation, EBL is the most common infectious disease in cattle, accounting for 67.1% of all infectious diseases diagnosed in cattle [1]. Recently, in some regions of Russia, there has been a slight decrease in the number of EBL- and BLV-infected cattle. However, the epizootic situation in 28 of the 60 regions of Russia has not improved according to statistics from 2019 [2].

Following BLV infection, three stages in the disease progression can be identified: (i) most BLV-infected cattle remain clinically asymptomatic through their lifetime, (ii) approximately one-third of them develop persistent lymphocytosis (PL) characterized by a permanent increase in the number of circulating peripheral IgM+ B cells, and (iii) less than 5% of infected cattle develop B-cell leukaemia/lymphosarcoma after long latency periods (4–10 years) [3].

BLV is transmitted horizontally. Many common farm practices have been implicated in viral transmission, including tattooing, dehorning, injections, and blood collection. Transmission may occur transplacentally from an infected dam to the foetus, intrapartum by contact with infected blood, or postpartum from the dam to the calf through ingestion of infected milk. Moreover, close contact is thought to be a risk factor for transmission, since cows with PL are at greater risk of passing BLV infection to their calves and other cows [4,5,6].

The propagation of BLV in BLV-infected cattle depends mainly on the mitotic division of infected cells (clonal expansion) [7]; it is also thought that a small population of the BLV-infected cells produce infectious virions [8]. The level of viral propagation in BLV-infected cattle can be assessed by the proviral load (PVL), defined as the number of proviral copies in blood cells [9]. The PVL in PL is significantly higher than in asymptomatic BLV-infected cattle [10,11]. PVL can remain stable over extended periods of time or increase with progression of the disease. High PVL is a risk factor for developing EBL [11,12]. 

The BLV genome consists of *gag*, *pol* and *env* structural genes and a region X, which contains several open reading frames for Tax, Rex, R3 and G4 regulatory proteins [8]. The BLV genome is flanked at both 5′ and 3′ ends by long terminal repeat (LTR) sequences [13,14]. The susceptibility to disease is associated with specific alleles of the major histocompatibility complex (MHC) class II BoLA-DRB3 gene [15,16]. However, there is little data available on the factors controlling B-cell mitosis in BLV-infected cattle. The important question remains: what is the driving force of the clonal expansion process? Based on the widely described properties of the BLV-encoded Tax protein [17,18], it is assumed that this protein triggers cell proliferation. Additionally, the selective growth advantage of the infected cells is spurred by viral non-coding RNAs (i.e., lncRNAs and miRNAs) [9,19,20,21]. The BLV provirus constitutively expresses antisense transcripts AS1-S/L and AS2 in all leukemic and asymptomatic individuals, which may play a role in the regulation of BLV [21]. Additionally, the seed of one BLV miRNA (BLV-miR-B4) is similar to that of host miR-29 associated with B-cell neoplasms [22,23]. Consistent with the notion that BLV-miR-B4 contributes to BLV-associated tumorigenesis, the main pathway driven by BLV miRNAs pertains to proliferation of B lymphocytes [9]. Moreover, the miRNAs interfere with host immunity and are associated with reduced expression of genes involved in B-cell differentiation [24].

Given the alleged roles of Tax and the miRNAs in BLV replication and pathogenesis, little is known about their sequence variation. The genetic variability of the *tax* gene may determine the clinical course of the disease [25,26,27]. The naturally occurring Tax L(233)P substitution correlates with leukemogenicity in BLV-infected cattle [28]. A BLV isolate with E(303)K mutation is deficient for infectivity in vivo, resulting in reduced transcriptional activity of the LTR promoter in vitro and inducing the silent BLV phenotype in a BLV-induced B-cell tumor [29,30]. Another substitution, E(51)G, located in the putative zinc finger domain of Tax, is associated with increased white blood cell (WBC) count in BLV-infected cattle [31]. On the other hand, differences in WBC counts may depend on the presence of single nucleotide polymorphisms (SNPs) in BLV pre-miRNA genes (pre-miRNA-B2, -B3, -B4 and -B5) [32]. 

In addition to Tax and miRNAs, sequence variations in the BLV 5′-LTR also affect viral replication. LTR is composed of three regions: U3, R and U5. The U3R region of the 5′-LTR is involved in the control of both basal and Tax-dependent transcription of the BLV genome through its interaction with several cellular transcription factors (reviewed in [33]). A natural substitution within the TATA box T(175)C is associated with viral productivity in vitro and BLV transmissibility [34,35,36]. 

Three regions of the BLV provirus (Tax, miRNA and LTR) have thus been associated with PVL, lymphocyte counts or pathogenesis. In this paper, we investigated the correlation between these sequence variations and disease outcome in naturally BLV-infected cattle with low and high lymphocytosis in Russia.

## 2. Results

### 2.1. Selection of Cattle with High and Low Lymphocytosis 

The analyzed population of 44 samples had a variate (bimodal in shape) distribution of WBC counts (Figure S1). The cluster analysis classified and identified the two groups of blood samples, where 22 000 WBCs per μL was set as the threshold between the two groups (Table S1A,B). Therefore, the number of leukocytes, <22 000 WBCs and ≥22 000 WBCs per μL, were considered to be low and high persistent lymphocytosis (PL), respectively. Finally, WBC count intervals ranged from 11 000 to 21 000 and from 22 000 to 42 000 WBC/μL, respectively, for low and high PL. 

### 2.2. Variation among Tax Amino Acid Sequences

Out of the 48 genomic DNA samples used for PCR amplification of the *tax* gene, 34 amplicons were successfully sequenced. The *tax* sequences were translated and aligned in Geneious Pro to perform a standard protein alignment (Figure S2). The Tax sequences revealed 97.3% averaged pairwise identity. A consensus sequence calculated from multiple alignment was used to determine amino acid variations for individual strains. Missense mutations from the consensus were found in 33 variants. The same changes were observed in variants derived from the same herds, as well as different herds and breeds. The most frequent changes within Tax functional domains and epitopes included T(52)I in Zn finger domain; Y(257)C in multifunctional domain; L(278)I in B-cell epitope; and T(167)I, L(173)P, R(183)K in CD8+ CTL epitope, which encompass the leucine-rich activation domain and transcriptional activation domain within this epitope, respectively. Subsequently, all Tax sequences were grouped into fifteen variants, named successively from Tax_A to Tax_O, representing distinct mutational patterns with respect to known functional domains and epitopes. The missense mutations in Tax are shown in Table 1. Phylogenetic analysis showed that the Tax_A–K and Tax_O variants belong to genotype 4, and the remaining variants, Tax_L–N, belong to genotype 7 (Figure S3).

### 2.3. Identification of Single Nucleotide Polymorphisms in BLV miRNAs and Promoter Elements

Out of 48 genomic DNA samples used for PCR amplification of the miRNA encoding region, 40 amplicons were successfully sequenced. The miRNA region sequences showed 98.4% averaged pairwise identity. A consensus sequence built for the multiple alignment was used to determine nucleotide variations within pre-miRNA-B genes and their promoter elements. In addition to pre-miRNA-B1, single nucleotide polymorphisms (SNPs) were identified along all pre-miRNA-B genes (Figure S4). The most frequent changes included A(154)G in BLV-mir-B2-3p, C(249)G in BLV-mir-B3-5p, A(310)del in BLV-mir-B4-5p, A(357)G in BLV-mir-B4-3p, A(462)G in BLV-mir-B5-5p and GA(497–498)AG in BLV-mir-B5-3p. Additionally, 18 putative A-box-1,-2,-3, six putative B-box and seven terminator sequences were identified (Figure S4). Most pre-miRNA genes contained 3 overlapping A boxes before, within, and downstream of the 3p arm, and they indicated a YRR(N:4-8)RR motif. B-boxes were most often located behind the 3p arm or between the pre-miRNA genes and consisted of varied sequence motifs as follows: (1) GTTCGAAC, (2) RGTTCGCG, (3) RGWTAAGAC, (4) RGTTCGAATC, (5) GTTGCRCAC and (6) RGGTTGTG. The promoter and terminator elements, identified based on the consensus sequence analysis, were rather stable for all variants. Averaged pairwise identity values between promoter and terminator elements (98.2%) and the pre-miRNA genes (98.9%) were comparable. Moreover, the “seed” regions (nucleotides 2–7) that are responsible for targeting miRNAs to mRNA transcripts were largely conserved, with the exception of the previously identified SNPs in BLV-miR-B5-3p (ref BLV FLK strain). Subsequently, all sequences were divided into 11 variants, based on their polymorphisms in pre-miRNA-B genes and promoters, and were named miRNA_A to miRNA_K. The SNP characteristics for each miRNA variant are shown in Table 2. Phylogenetic analysis showed that the miRNA_B–F and miRNA_H–K variants belong to genotype 4, and the remaining variants, miRNA_A, G, belong to genotype 7 (Figure S5). 

### 2.4. Identification of SNPs in BLV LTR 

Out of 48 genomic DNA samples, 41 full-length LTRs were successfully amplified and sequenced. The LTR sequences shared 98.5% average pairwise identity. A consensus sequence calculated from multiple alignment of all LTR sequences was used to determine nucleotide polymorphisms within each individual sequence (Figure S6). The same nucleotide sequence variations were observed between the variants derived from the same or different cattle herds and between different cattle breeds. The changes occurred through the whole LTR sequence, both within and outside enhancers and regulatory elements. The most frequent changes observed in regulatory elements included the G(−133)A/C in TRE2 and A(−4)G in 7-methylguanosine cap (CAP site) substitution within the U3 subregion; and A(+150)G in Box A, C(+160)T in downstream activator sequence (DAS), T(+161)C in DAS, C(+183)T in DAS and C(+187)A in Box C substitutions within the R subregion. The interferon regulatory factor (IRF) binding site located in the U5 subregion was conserved for all strains. The motif ten element (MTE)/downstream promoter element (DPE) for RNAP II antisense transcription of long non-coding RNAs (AS1-S/L and AS2) within the U5 subregion indicated 100% sequence identity. The TFIIB recognition element (BRE) (5’-GGCGCCC-3’) in U5, besides the two SNPs in PL4 cattle located on the first and third positions of the motif, 5’-AGAGCCC-3’, indicated maximal sequence identity (Figure S6). Next, LTR sequences were divided into 13 variants, labeled from LTR_A to LTR_M, based on their mutations with respect to the regulatory elements. SNPs present in particular LTR variants are shown in Table 3. Phylogenetic analysis showed that the LTR_A–K variants belong to genotype 4, and the remaining variants, LTR_L, M, belong to genotype 7 (Figure S7).

### 2.5. An Association between BLV Sequence Variants and the Level of Persistent Lymphocytosis 

**Figure 1:**
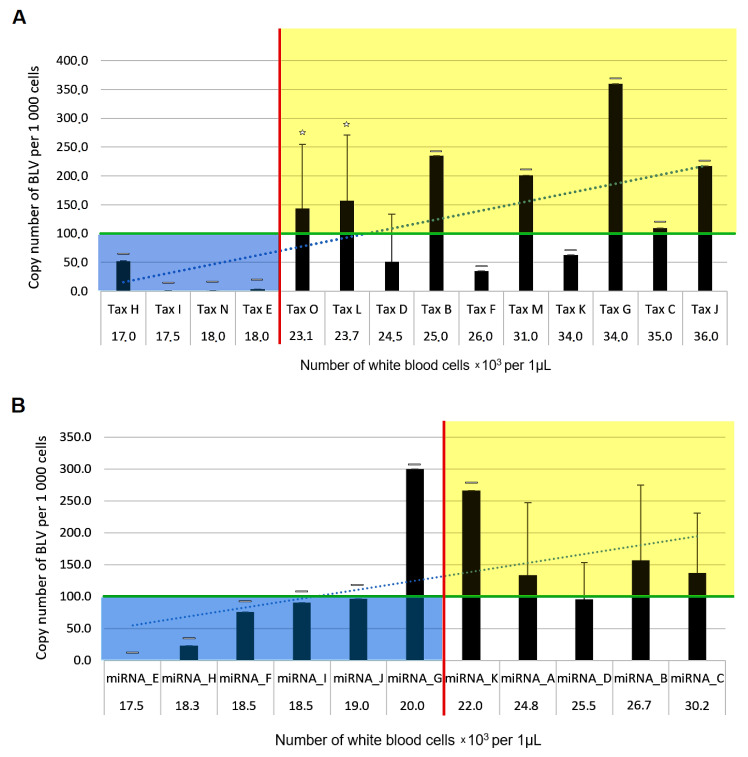

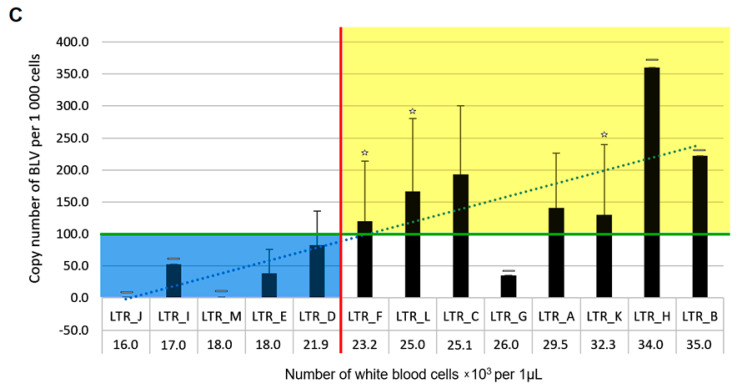
Correlation between proviral copy numbers and white blood cell (WBC) counts for Tax (**A**), miRNA (**B**) and LTR (**C**) variants classified as low and high persistent lymphocytosis (PL). The averaged values of white blood cell count per microliter of blood were sorted on the x-axis from the lowest to the highest. The red line indicates the threshold value between group I with low PL (WBC < 22 000 leukocytes/μL) and group II with high PL (WBC count ≥ 22 000 leukocytes/μL). The green line is the threshold value between group I (proviral load (PVL) below 100 copies per 1 000 cells) and group II (PVL > 100 copies/1 000 cells). Variants classified to the group I (with low PL) are in the blue field. Variants classified to the group II (with high PL) are in the yellow field. All data are expressed as mean ± SD. A horizontal dash (-) used above the bars indicates that a single isolate constitutes a group. (**A**) Classification of the 14 Tax variants in low and high PL groups. Asterisk (*) used above the bars indicates significant difference in PVL values between Tax_O group and Tax_H, I, N, E variants assigned to the low PL group (Mann–Whitney U Test *p* = 0.04236) or between Tax_L group and Tax_H, I, N, E variants (Mann–Whitney U Test *p* = 0.01208). The blue dashed line indicates the trend line, Rs = 0.367. (**B**) Classification of the 11 miRNA variants as low and high PL. There was no statistically significant difference in PVL values between miRNA_A, miRNA_D, miRNA_C or miRNA_B groups and miRNA_E, H, F, I, J variants assigned to the low PL group (Mann–Whitney U Test *p* = 0.3734, *p* = 0.37828, *p* = 0.39358 and *p* = 0.3263, respectively). The blue dashed line indicates the trend line, Rs = 0.257. (**C**) Classification of the 13 LTR variants in low and high PL groups. Asterisk (*) indicates statistical significance of PVL values between LTR_F, LTR_L or LTR_K group and LTR_J, I, M, E, D variants assigned to low the PL group (Mann–Whitney U Test *p* = 0.04182, *p* = 0.01778, and *p* = 0.0548, respectively). The difference in PVL was close to being statistically significant for the LTR_C or LTR_A group and LTR_J, I, M, E, D variants assigned to the low PL group (Mann–Whitney U Test *p* = 0.11314 and *p* = 0.06057, respectively). The blue dashed line indicates the trend line, Rs = 0.594.

The WBC count and proviral load (PVL) were further used to evaluate the relationship between Tax, miRNA and LTR sequence polymorphisms. Samples were divided into two groups: group I, with a low white blood cell (WBC) count ranging from 12 000 to 21 000 leukocytes per μL and a proviral load below 100 copies per 1 000 cells; and group II with a high WBC count ranging from 22 000 to 42 000 leukocytes per μL and a proviral load above 100 copies per 1 000 cells. An average value of PVL and WBC count for each of the 15 Tax variants, 11 miRNA variants and 13 LTR variants was calculated (Figure 1A–C). 

Graph-based analysis enabled segregation of a majority of variants into the two previously established groups. Four Tax variants (Tax_E,_H,_I,_N), five miRNA variants (miRNA_E,_F,_H–J) and five LTR variants (LTR_D,_E,_I,_J,_M) were each assigned to group I (PVL < 100 copies/1 000 cells and WBC < 22 000 leukocytes/μL). Seven Tax variants (Tax_B,_C,_G,_J,_L,_M,_O), five miRNA variants (miRNA_A–D,_K) and six LTR variants (LTR_A,_B,_F,_H,_K,_L) were assigned to group II (PVL > 100 copies/1 000 cells and WBC count ≥ 22 000 leukocytes/μL). The remaining variants, i.e., Tax_D,_F,_K, miRNA_G and LTR_G, representing 13% of the total sample population, could not be classified anywhere since they had an uncorrelated number of leukocytes with the number of provirus copies. The variant A was associated with leukemia and characterized as Q(40)L and V(142)A in T-cell epitope.

The polymorphisms characteristics for the low PL group are shown in Table 4. 

The Tax variants from this group indicated R(43)K in Zn finger domain, F(178)Y, V(212)I, within and in proximity to the CTL epitope overlapped with the leucine-rich domain and I(246)T, F(249)L in the multifunctional domain (all mutation was identified in 1 out of 4 isolates, creating a low PL group for Tax). The miRNA variants were characterized by G(141)A in B2 pre-miRNA, A(420)H in putative B-box (identified in 2 out of 5 isolates, creating a low PL group for miRNA), C(239)T in BLV-mir-B3-5p, and A(467)G in BLV-mir-B5-5p and putative B-box (identified in 1 out of 5 isolates). LTR variants indicated substitutions as follows: G(−142)A in NF-kB-like protein, T(−65)C in GRE, T(−11)C in CAP site (each was detected in 1 out of 10 isolates, creating a low PL group) and A(−4)G in CAP site (in 7 out of 10 isolates).

The polymorphism characteristics for the high PL group are shown in Table 5. 

The Tax variants from this group indicated E(42)K and E(51)K in Zn finger domain (1 out of 23 isolates, 4.3%), Q(117)R, I(131)V, V(142)A in T-cell epitope (in 1 out of 23 isolates, 4.3%) and A(198)T in CD8+ T-cell epitope (in 1 out of 23 isolates, 4.3%) substitutions. The miRNA variants were characterized by A(154)G in BLV-mir-B2-3p (in 5 out of 34 isolates, 14.7%), C(249)G in BLV-mir-B3-5p (in 5 out of 34 isolates, 14.7%), A(310)del in BLV-mir-B4-5p, A(357)G in BLV-mir-B4-3p, A(462)G in BLV-mir-B5-5p and GA(497–498)AG in BLV-mir-B5-3p mutations (in 9 out of 34 isolates, 26.5%). LTR variants indicated substitutions as follows: G(−133)A/C in CRE2 (in 14 out of 30 isolates, 46.7%), T(−122)A in Ebox2 (in 3 out of 30 isolates, 10%), A(+150)C in BoxA (in 1 out of 30 isolates, 3.3%), C(+160)T (in 9 out of 30 isolates, 30%), C(+183)T in DAS (in 5 out of 30 isolates, 16.7%) and C(+185)T in Box C (in 1 out of 30 isolates, 3.3%) (Table 5). 

In addition, mutational analyses of the proviral Tax, miRNA and LTR sequences were conducted to determine if a correlation existed between the frequency of mutations and the PL stage. The Analysis of Tax sequence and its amino acid polymorphisms in low and high PL groups revealed four predominant mutations, I(214)T, T(221)S, L(233)P and S(281)P detected respectively in 65.2% (15/23), 43.5% (10/23), 47.8% (11/23) and 47.8% (11/23) of variants assigned to the high PL group (Figure S8A). The miRNA sequence analysis revealed that del(168)T and A(441)G mutations were both detected in 55.9% (19/34) of variants with high PL (Figure S8B). No significant differences between low and high PL groups (*p* > 0.05) with the above-described mutations were found (Table 6). On the other hand, LTR analysis revealed one relevant mutation, A(+187)C within the Box C of the DAS region, which was identified in 76.7% (23/30) variants with high PL (Figure S8C). A significant difference in detection of this mutation was found between variants with high and low PL (*p* < 0.05 for both) (Table 6).

## 3. Discussion

The characterization of new mutations in functionally important fragments of the BLV genome is an ongoing research area. To this extent, the pX region, LTR and envelope protein polymorphisms have been studied widely by sequencing the strains in BLV-infected cattle around the world. Only limited studies have shown the identified mutations with reference to the clinical stages in naturally infected cattle. 

In this study, 48 whole blood samples were collected from BLV infected cattle that developed persistent lymphocytosis (44 cows), lymphoma (one cow) and aleukemic form (3 cows). The proviral copy number was then determined for each sample. High proviral loads have been associated with EBL progression [37,38,39] and have been conventionally used in several studies as a determining index to forecast the progress of the disease, thereby justifying its use in our study. Two key parameters, the number of proviral copies (>100 copies per 1000 WBCs) and WBC count (≥22 000 leukocytes per µL), were considered significant and linked with the high PL stage. 

The full-length sequences of *tax* gene, miRNA encoding region and LTR were amplified using nested PCR and overlap extension approaches. All sequences generated in this study were deposited in GenBank. The ClustalW algorithm was used to perform multiple alignment—the gateway for further analysis of nucleotide and amino acid sequence variability. Despite the evidence in the literature of Tax, miRNA and LTR involvement in BLV pathogenesis [9,21,27,40,41,42,43] and the ample evidence of sequence variation within the Russian samples found in this study, we failed to identify specific Tax, miRNA or LTR signature sequences that could be clearly linked to different levels of PL. Nevertheless, some differences in the sequences corresponding to the groups of cattle with low and high lymphocytosis were observed. 

The amino acids at positions 213T and 220S adjacent to the CTL epitopes, and 233P and 281P next to multifunctional domain and B-cell epitope, respectively, predominated among Tax variants with high PL. In addition, individual Tax sequences derived from the group with high PL revealed several specific changes, including Q(117)R, I(131)V and V(142)A in the T-cell epitopes, A(198)T in CD8+ T-cell epitope, and Q(40)L, E(42)K, and E(51)K in the putative zinc finger domain. On the other hand, several Tax variants derived from the group with low PL indicated specific changes, including F(178)Y, V(212)I, I(246)T, F(249)L and R(43)K within and in proximity to the CTL epitope and overlapped with the leucine-rich domain, multifunctional domain and Zn finger domain. We therefore hypothesize that the above-mentioned polymorphisms may induce alteration of viral properties related to viral replication and pathogenesis through different stimulation of cell growth of BLV-infected cells and in the presence of a strong immune surveillance.

Interestingly, Zyrianova et al. identified the change E(51)G in the zinc finger motif of BLV strain derived from a cow with high lymphocytosis [31]. In our study, variants Tax_A,_B and _C, which contained Q(40)L, E(42)K, and E(51)K substitutions, displayed increased WBC count and BLV copy numbers. The N-terminal zinc finger domain is an integral element of Tax structure and is involved in the Tax transactivation of viral and cellular promoters. Tax activates LTR-directed transcription of viral genes by activating and recruiting cellular transcription factors, including members of the CRE-binding/activating transcription factor (CREB/ATF) family, to three imperfectly conserved 21 base pair repeats located in the LTR. Tax also activates other cellular transcription factors, such as NF-κB and AP-1, resulting in global changes in gene expression that favor cell growth, activation and survival [44,45]. In BLV, amino acids at positions 30C, 33C, 50C and 53H within the zinc finger domain are essential to the Tax transactivation function. The mutants at these locations lost their transactivating capacity; however, they were still able to induce leukemogenesis [46]. Proper folding of Tax may play a role in oligomerization and association with cellular transcription factors, determining the specificity of Tax transactivation. 

Cellular immunity against BLV contributes to the suppression of BLV replication. Nevertheless, the persistence of antibodies to viral proteins in BLV-infected cows indicates a continuous, although low level, of viral gene expression. The number of CD8+ T cell epitopes appeared to be correlated with the proviral load in BLV-infected cattle. Besides the structural proteins (envelope gp51 and capsid p24), Tax is the target of a strong CD8^+^ cytotoxic T lymphocyte (CTL) and CD4 helper T lymphocyte response to BLV [47,48]. Bai et al. suggested that Tax-specific CTLs are induced only in cows with high (>585 copies per 1 000 cells) proviral loads, since the Tax expression is weak compared with the expression of the envelope proteins [48]. In the group of high PL cattle, four Tax variants were characterized with Q(117)R, I(131)V and V(142)A changes in the T-cell epitopes [49] and an A(198)T change in CD8+ T-cell epitope [48]. Of note, the V(142)A change was found to have occurred in the two strains in this study with high WBC counts and proviral copy numbers as well as four more strains from Russia (Acc No. MN072355) and Iran (Acc No. MG204549.1, MG204544.1, MG204542.1), which all derived from PL cattle [31,49]. Mutations in HTLV-1 Tax CTL epitopes have been associated with escape from anti-Tax CTL response in vivo, and Niewiesk et al. suggested that this may contribute to disease pathogenesis [50]. Therefore, it is possible that the mutations found here may somehow promote virus propagation in the host. On the other hand, the Tax variants from the low PL group exhibited F(178)Y and V(212)I, within and in proximity to the CTL epitopes. Further work to investigate the functionality of these mutations in these sequences is ongoing. 

The changes within multifunctional domain I(246)T, F(249)L and leucine-rich domain F(178)Y identified among Tax variants in the low PL cattle group may be functionally significant as these domains are involved in the activation of BLV LTR promoter and the expression and propagation of BLV and/or cellular genes [45].

The miRNA locus was maintained in all 41 BLV variants, further supporting their contribution to persistent lymphocytosis and tumorigenesis [19,20]. The nucleotides at positions 168T adjacent to the terminator element and putative A-box and 441G predominated among miRNA variants with high PL; however, these mutations appeared to be functionally insignificant for BLV replication. Importantly, the A-box, B-box, and transcription terminator elements required for BLV pre-miRNA expression, as well as the pre-miRNA structures, were maintained. These observations indicate that the BLV pre-miRNAs from these strains were transcribed and processed into miRNAs as previously described [20,51]. The miRNA sequences were largely conserved, particularly in the “seed” region (nucleotides 2–7) that targets the RNA−induced silencing complex (RISC) to mRNA transcripts for silencing. This indicates that the miRNAs from these strains regulate the same mRNA transcripts, implying that they are functional. A notable exception to this is the strains containing the miRNA_A and miRNA_G variants (classified to genotype 7), in which the “seed” region of BLV-miR-B5-3p contains a GA to AG polymorphism. Interestingly, this version of BLV-miR-B5-3p is also present in previously identified BLV isolates BL3.1 (Acc No. LC436098), FLK-BLV913 (Acc No.EF600696) as well as strains with Acc No. MF580995, LC080658, AF257515, Poland_3 (Acc No. MW470848) classified to the genotypes G1, G10, G6, G2 and G8. The increased frequency of this polymorphism in BLV strains suggests that these nucleotide positions tolerate mismatches in the target and/or that there is a yet-to-be-identified biologically relevant function for this polymorphism. Interestingly, both miRNA_A and miRNA_G variants, which contain this polymorphism, display increased copy numbers of BLV genomes (Figure 1B), suggesting that the GA to AG polymorphism in BLV-miR-B5-3p may promote BLV replication and integration. Therefore, further functional analysis of this polymorphism is warranted. 

Comparison of LTR variants associated with low and high lymphocytosis revealed some differences. The respective purine at position −4A within CAP site and pyrimidine at +187C in box C of the DAS region predominated among variants assigned to high PL. In deltaretroviral LTR sequences, the CAP site overlaps with the TATA box-binding protein site and the RNAPII-binding promoter region. In this arrangement, the two elements may contribute to core promoter activity. It is not, however, clear if the mutations within this region play a role in abnormal initiation of transcription. Unfortunately, a functional consequence of the A to C transversion in box C (+187) has not been explored before. 

Additionally, an individual LTR variant derived from the group with low PL was characterized with specific SNPs in the U3 region, including inter alia G(−142)A in NF-kB like protein site. Interestingly, the mutant AG(−142/−143)CT in the putative NF-kB site showed less than 20% of the wild-type LTR promoter activity in in vitro study [52]. On the other hand, individual LTR variants associated with the group of samples from high PL cattle harbored specific SNPs in the U3 region, including G(−133)A/C in CRE2 and T(−122)A in E-box-2. 

The promoter transcriptional activity of the 0222GD_K–P strain (Acc No. MH423661.1) derived from infected Polish cows, which was characterized by the G(−133)A substitution in CRE2, was previously analyzed (unpublished data). As a result, the variant revealed a slightly increased promoter activity (1.5-fold enhancement, *p* < 0.05) in comparison with the reference pLTR-WT, 344 strain, in in vitro study. The T(−122)A substitution in Ebox2 can significantly increase the LTR basal promoter activity [53]. It is known that E-box elements in the BLV genome act as repressors by binding directly to the CRE enhancers [54]. Mutations found here might disrupt the binding site for the transcription repressor and finally contribute to increased promoter activity. For example, the substitution G(−124)T in the AP4-2 transcription factor binding sequence gave rise to the strongest promoter function in vitro, 14 times higher that of the wild-type LTR [52]. 

The U3R region of the BLV 5′-LTR is involved in the control of both basal and Tax-dependent transcription of the BLV provirus through its interaction with several cellular transcription factors. Therefore, mutations affecting the promoter nucleotide sequence might drive potential differences in the biological properties of diverse BLV strains [34,36]. In the early stages of infection and prior to seroconversion (when BLV is transferred to a new organism) variants with a stronger LTR promoter may enhance viral gene expression and influence the course of the infection. However, during lymphocytosis, active viral transcription is almost undetectable and does not contribute significantly to the observed PVL [55,56,57,58]. This is caused by cytotoxic T lymphocytes, which play a central role in the protective immune response against BLV [59]. During the chronic stage, BLV replicates through the clonal expansion of BLV-infected cells, which is clearly associated with an increase in viral load. The BLV genetic variation at this stage of infection is generated by somatic mutations occurring within the cellular as well as the proviral sequences [60,61]. Even if mutations are often under strong negative selection, they may contribute to avoiding CTL recognition, thereby promoting leukemogenesis [62].

We hypothesize that the above-described differences in Tax, miRNA and LTR sequences for BLV strains derived from cows with low and high lymphocytosis may suggest the existence of quasispecies. Such populations of proviral clones in individual cows with lymphocytosis have been identified for Tax and miRNA genes (so-called alleles) by Zyrianova et al. [31,32]. It is noteworthy to mention that the presence of HTLV-1 quasispecies, as multiple infection, was also suggested in HTLV-1, which belong together with BLV to the *Deltaretrovirus* genus [63,64]. Indeed, some of the clones would be more dominant than others and potently modulate viral replication and course of disease in vivo. In this study, the PCR amplicons were sequenced using the Sanger dideoxy sequencing method, where the obtained sequencing reads are an average of all DNA present in the PCR mixture. Unfortunately, this approach is insufficient to clearly identify potential quasispecies. The obtained sequences therefore represent major clones. Further research of this phenomenon is warranted. 

## 4. Materials and Methods

### 4.1. Sample Collection and Preparation

Blood samples were obtained from 44 cattle coming from four farms in the Stavropol and Krasnodar regions of Russia, representing three cattle breeds: Holstein-Friesian (21), Ayrshire (13) and Red cattle (10). Blood samples from cattle naturally infected with BLV were selected from a collection of samples taken by local veterinary services as part of the EBL monitoring program. The samples were sent to the laboratory of Novosibirsk State Agrarian University in Russia for this study. All animals were classified as BLV-infected by the agar gel immunodiffusion test (BIOK, Kursk, Russia), and all showed persistent lymphocytosis (PL). Additionally, one cow of the Holstein-Friesian breed showed clinical symptoms of lymphoma, and three cows were aleukemic (AL) (Table 7). 

White blood cells (WBCs) were counted under a microscope using a Goryaev chamber, and differential WBC count was obtained from Romanovsky-Giemsa-stained blood smears in the laboratory of Novosibirsk State Agrarian University in Russia. The PL stage of BLV infection was evaluated according to the lymphocyte count (cells per μL) of each cow [65]. Additionally, to differentiate aleukemic from nonaleukemic cattle, the WBC count reference interval (value of 13 400 WBC count per μL) was used [66]. A strong correlation was observed between lymphocyte count and WBC count (r = 0.965, *p* < 0.0000001) (Figure S9). For further analyses, the WBC parameter was used. 

The EDTA-treated whole blood samples were used for extraction of genomic DNA with a DNA-sorb-B Nucleic acid Extraction Kit (AmpliSens, Moscow, Russia), according to the manufacturer’s instructions. The DNA samples were lyophilized and transported to the National Veterinary Research Institute in Puławy, Poland for further study. 

### 4.2. H3F3A Gene Quantitative Real-Time PCR Development

To measure the BLV proviral copy number per 1 000 cells, a new real-time PCR (qPCR) assay for quantification of bovine histone H3 family 3A (H3F3A) housekeeping gene was developed. The bovine-specific primers and probe were designed using the “Design new Primers” tool in Geneious Prime and synthesized in Genomed (Warsaw, Poland). The primers and probe sequences and characteristics are shown in Table S2. The target region from the H3F3A gene was amplified and cloned into the pCR4-TOPO vector (Invitrogen Life Technologies, Carlsbad, CA, USA). Plasmid DNA (pDNA) was linearized by digestion with PstI, and pDNA concentration was measured using a nanophotometer (Implen GmbH, Munich, Germany). The copy number of pDNA was calculated based on the linear plasmid concentration and the molecular weight. Ten-fold serial dilutions of the pDNA from 10^2^ to 10^6^ copies per μL were used as standard in all qPCR assays to calculate numbers of H3F3A copies in each DNA template. The amplification by qPCR was accomplished in a 25 μL final-volume-containing mixture of 12.5 μL 2× QuantiTect Multiplex PCR NoROX master mix (Qiagen AG GmbH, Hilden, Germany), 0.4 μM of each primer (Genomed, Warsaw, Poland), 0.2 μM probe, and extracted genomic DNA. The reactions were performed in the Rotor-Gene Q cycler (Qiagen) using an initial denaturation step and polymerase activation at 95 °C for 15 min, followed by 40 cycles at 94 °C for 60 s and at 60 °C for 60 s. Two technical repeats were run for each DNA sample, and the average of two PCR repetitions was used for the further study. A standard curve was constructed, and the results agreed with the Minimum Information for Publication of Quantitative Real-Time PCR Experiments (MIQE) criteria (the R^2^ value was above 0.98, the slope of standard curve was between 3.1 and 3.6, and the PCR efficiency was above 90%) [67]. 

### 4.3. Quantification of BLV Provirus Copy Number

The number of provirus copies per 1 000 cells was calculated as follows: (copy number of BLV pol)/(copy number of H3F3A/2) × 1 000 cells. The qPCR for the BLV pol gene was performed according to a previously published procedure [68]. PVLs < 100 and > 100 copies per 1 000 cells were considered to be relatively low PVL and high PVL, respectively. To support this division, Jimba et al. showed that the syncytia formation correlated strongly with a BLV PVL of over 10 000 copies/10^5^ cells [12,37]. BLV provirus was detected in milk samples of infected cows only when the PVL in the blood samples exceeded 10 000 copies/10^5^ cells [37]. The protein arginine-*N*-methyltransferase (PRMT5), which may affect BLV infected cows’ progression from the asymptomatic stage to the lymphoma stage, was significantly upregulated in BLV-infected cattle with PVL > 10 000 copies/10^5^ [69]. 

### 4.4. Amplification of LTR, pre-miRNA and Tax Gene Sequences

The full-length LTR and *tax* gene were amplified respectively by OE-PCR and nested PCR, using the oligonucleotide primers, Q5 High-Fidelity DNA Polymerase and Q5 Reaction Buffer (New England BioLabs, Ipswich, MA, USA) as previously described [36]. The miRNA coding region was amplified by nested PCR amplification using two sets of primers as described in Table S2. Both rounds of PCR amplification were performed using PrimeSTAR GXL DNA Polymerase and Prime STAR GXL buffer (Takara Bio, Kyoto, Japan). Thermal cycling conditions for the first round of PCR were as follows: 2 min at 98 °C, 35 cycles (15 s at 98 °C, 25 s at 55 °C, 3 min at 68 °C) and 10 min at 72 °C. For the second round, thermal cycling conditions were 2 min at 98 °C, 35 cycles (15 s at 98 °C, 25 s at 70 °C, 1 min at 68 °C) and 10 min at 72 °C. The PCR products were separated and analyzed by electrophoresis on 1.5% agarose gel containing SimplySafe (EURx, Gdansk, Poland), diluted 1:10 000 in 1× TAE buffer. All PCR products were purified using a NucleoSpin Extract II Kit (Marcherey Nagel GmbH & Co, Hamburg, Germany) and sequenced in Genomed SA Company (Warsaw, Poland) using a 3730xl DNA Analyzer (Applied Biosystems, Foster City, CA, USA) and a Big Dye Terminator v3.1 Cycle Sequencing Kit. 

### 4.5. Sequence Data Analysis 

Raw sequence data were proofread in Geneious Prime 2019.0.3 (Biomatters Ltd, Auckland, New Zealand). For each strain a consensus sequence was determined and deposited in the GenBank database under accession numbers MW256513–MW256627. The Tax amino acid sequences, translated according to the IUPAC amino acid code, and nucleotide sequences of LTR and pre-miRNAs were aligned using the ClustalW algorithm, implemented in Geneious Prime. Genetic distance analysis between newly obtained sequences and reference sequences from other countries were calculated using the Maximum Composite Likelihood model in MEGA X. For all fragments, the Maximum Composite Likelihood model and Bootstrap replications (1 000) were applied in MEGA X to infer a phylogenetic tree according to the Neighbor-joining method [70]. Transcription factor binding site (TFBS) modifications related to specific mutations in LTR were analyzed in the Geneious Prime plugin, based on the EMBOSS 6.5.7 tool tfscan [71]. The EMBOSS suite of fuzznuc applications for detection of miRNA patterns was used [72]. For B-box sequences, the GTTCNANNC, GGTTSGNG, RGTTCRANNCC and GKWCAAGTC motifs were used. For A-box-1,2,3 sequences, the TGRNNNNNNGR, TRGNNNNNNGR and TRGNNNNNGR motifs were used according to Kincaid et al. [20,73,74]. 

### 4.6. Statistical Analysis

To measure the strength of the association between lymphocyte count and WBC count, the Pearson’s correlation coefficient (r) was used. The distribution of WBC counts from the collected blood samples was analyzed using a histogram plot. To identify true groups among WBCs in collected blood samples, the k-means algorithm was used. The Euclidean distances between clusters were computed. The difference in pairwise identity between promoter and terminator elements and the whole miRNA encoding region were calculated using the Student’s t-test. Chi-Square and Mann–Whitney tests were used where a *p* value of <0.05 was considered to be significant. All the statistical analyses were performed using STATISTICA Data Miner (StatSoft, Tulsa, OK, USA).

## 5. Conclusions

In this study, we characterized Tax, miRNA and LTR sequences from BLV-infected cows displaying varying levels of PL. Defective proviruses that may play a role in EBL pathogenesis were not identified. Molecular characterization of these regions showed that the genomic variability of these viruses was minimal. A statistically significant association was observed between the A(+187)C polymorphism in DAS region in LTR and high PL. Any mutation in Tax and miRNA sequences could be associated with specific high PL manifestations. Although some strains from the high PL group had point mutations within important genomic regions, these variations were specific only for this group. Further studies are necessary to verify the functional consequences of these polymorphisms.

Overall, the identified changes were segregated in accordance with the geographical origin of the strains rather than the PL stage. Phylogenetic analysis of Tax, miRNA and LTR regions showed that BLV could be grouped into two molecular genotypes (genotypes 4 and 7 are common in Russia), but a clear association between genotypes and the PL level was not found. 

## Figures and Tables

**Table 1:** Variations in 15 representative Tax sequences from bovine leukaemia virus (BLV) isolates. All indicated variations were compared with the consensus sequence shown in Figure S1.

Variant Name	Position	Amino Acid	Domain	Respective Number of Cattle	Genotype
Tax_A	40	Q -> L	Zn finger domain	LE48	4
	142	V -> A	T-cell epitope		
	233, 281	L -> P, S -> P	-*		
Tax_B	42	E -> K	Zn finger domain	PL33	4
	140	N -> I	T-cell epitope		
	142	V -> A	T-cell epitope		
	233, 281	L -> P, S -> P	-		
Tax_C	51	E -> K	Zn finger domain	PL42	4
	131	I -> V	T-cell epitope		
	186	I -> V	CTL epitope & Leucine-rich domain		
	69, 102, 214, 221, 228	T -> M, V -> I, I -> T, T -> P, E -> A	-		
Tax_D	52	T -> I	Zn finger domain	PL20, PL30, PL32, PL44	4
	183	R -> K	CTL epitope & Leucine-rich domain		
	278	L -> I	B-cell epitope		
Tax_E	52	T -> I	Zn finger domain	PL13	4
	140	N -> K	T-cell epitope		
	183	R -> K	CTL epitope & Leucine-rich domain		
	278	L -> I	B-cell epitope		
	69	T -> M	-		
Tax_F	52	T -> I	Zn finger domain	PL35	4
	124	S -> F	T-cell epitope		
	183	R -> K	CTL epitope & Leucine-rich domain		
	278	L -> I	B-cell epitope		
	2, 22, 73	S -> A, N -> S, R -> Q	-		
Tax_G	52	T -> I	Zn finger domain	PL41	4
	183	R -> K	CTL epitope & Leucine-rich domain		
	245	A -> T	Multifunctional domain		
	278	L -> I	B-cell epitope		
	235	Q -> R	-		
Tax_H	178	F -> Y	CTL epitope & Leucine-rich domain	PL5	4
	246	I -> T	Multifunctional domain		
	249	F -> L	Multifunctional domain		
Tax_I	245	A -> T	Multifunctional domain	PL9	4
	102, 214, 221	V -> I, I -> T, T -> P	-		
Tax_J	117	Q -> R	T-cell epitope	PL45	4
	102	V -> I	-		
Tax_K	141	L -> S	T-cell epitope	PL40	4
	186	I -> V	CTL epitope & Leucine-rich domain		
Tax_L	167	T -> I	CTL epitope & Leucine-rich domain	PL14, PL15, PL21, PL25, PL27, PL28, PL36, PL39, PL46	7
	173	L -> P	CTL epitope & Leucine-rich domain		
	257	Y -> C	Multifunctional domain		
	2, 69, 95, 214, 221, 233, 281	S -> A^5/9^ §, T -> A, A -> T, I -> T^8/9^, T -> S, L -> P, S -> P	-		
Tax_M	167	T -> I	CTL epitope & Leucine-rich domain	PL38	7
	173	L -> P	CTL epitope & Leucine-rich domain		
	198	A -> T	CTL epitope		
	257	Y -> C	Multifunctional domain		
	69, 95, 198, 214, 221, 227, 233, 281	T -> A, A -> T, A -> T, I -> V, T -> S, S ->T, L -> P, S -> P	-		
Tax_N	43	R -> K	Zn finger domain	PL10	7
	173	L -> P	CTL epitope & Leucine-rich domain		
	257	Y -> C	Multifunctional domain		
	2, 69, 95, 212, 221, 233, 281	S -> A, T -> A, A -> T, V -> I, T -> S, L -> P, S -> P	-		
Tax_O	102, 214, 221	V -> I^7/9^, I -> T^6/9^, T -> P ^6/9^	-	PL17, PL18, PL19, PL24, PL26, PL29, PL31, PL37, PL47	4

Single letter codes for the 20 amino acids are in accordance with IUPAC. Abbreviations: *, a site with no functional domain determined; §, five out of nine strains have the S -> A substitution within variant Tax_L; CTL, Cytotoxic T lymphocytes.

**Table 2:** Variations in 11 representative miRNA sequences derived from cattle BLV isolates. All the indicated variations were compared with the consensus sequence shown in Figure S2.

Variant Name	Position	Nucleotide	miRNAs	Respective Number of Cattle	Genotype
**miRNA_A**	84, 171, 195, 210, 212	A -> G, A -> G, G-> A, G-> A, T->A,	-^Գ^	PL8, PL14, PL15, PL25, PL28, PL36, PL38, PL39, PL46	7
	310	A -> del *	BLV-mir-B4-5p		
	342	G -> A	B4 pre-miRNA, putative A-box		
	357	A -> G	BLV-mir-B4-3p		
	373, 392, 453	G -> A, G -> A, C -> T	-		
	462	A -> G	BLV-mir-B5-5p		
	497–498	GA -> AG	BLV-mir-B5-3p		
**miRNA_B**	168	T -> del^14/15^	-	AL2, AL3, PL11, PL17, PL23, PL24, PL26, PL29, PL31, PL37, PL40, PL41, PL42, PL45, PL47	4
**miRNA_C**	106	G -> A^4/5^	-	PL5, PL12, PL33, PL43, LE48	4
	154	A -> G	BLV-mir-B2-3p		
**miRNA_D**	249	C -> G	BLV-mir-B3-5p, putative A-box	PL21, PL30, PL35, PL44	4
**miRNA_E**	141	G -> A	B2 pre-miRNA	PL9	4
	168	T -> del	-		
**miRNA_F**	141	G -> A	B2 pre-miRNA	PL18	4
	168	T -> del	-		
	171	A -> G	putativeA-box		
	91, 300	A -> G, A -> G	-		
**miRNA_G**	64, 67, 84, 171, 195, 210, 212	G -> A, A->T, A -> G, A -> G, G -> A, G -> A, T -> A	-	PL27	7
	310	A -> del	BLV-mir-B4-5p		
	341	C -> T	putative A-box		
	342	G -> A	B4 pre-miRNA, putative A-box		
	357	A -> G	BLV-mir-B4-3p		
	373, 392, 453	G -> A, G -> A, C -> T	-		
	462	A -> G	BLV-mir-B5-5p		
	497–498	GA -> AG	BLV-mir-B5-3p		
**miRNA_H**	97, 168	A -> G, T -> del	-	PL16	4
	420	A -> G	putative B-box		
**miRNA_I**	168	T -> del	-	PL19	4
	420	A -> G	putative B-box		
	467	A -> G	BLV-mir-B5-5p, putative B-box		
**miRNA_J**	107	A -> G	-	PL20	4
	239	C -> T	BLV-mir-B3-5p		
	249	C -> G	BLV-mir-B3-5p, putative A-box		
**miRNA_K**	181	G ->A	putative B-box	PL32	4
	249	C -> G	BLV-mir-B3-5p, putative A-box		

Abbreviations: *, deletion; ^Գ^, a site with no miRNA and regulatory element determined.

**Table 3:** Variations in 13 representative BLV long terminal repeat (LTR) sequences derived from cattle isolates. All the indicated variations were compared with the consensus sequence shown in Figure S3.

Variant Name	Position	Nucleotide	Regulatory Element	Respective Number of Cattle	Genotype
**LTR_A**	−133	G -> A	TRE2	PL12, PL22, PL33, LE48	4
	+5	C -> T	putative GR & PR site		
	+183	C -> T	DAS		
**LTR_B**	−133	G -> A	TRE2	PL43	4
	+5	C -> T	putative GR & PR site		
	+150	A -> C	Box A (DAS)		
	+183	C -> T	DAS		
	+185	C -> T	Box C (DAS)		
**LTR_C**	+187	C -> A	Box C (DAS)	AL1, AL2, PL23, PL26, PL31, PL37, PL45	4
**LTR_D**	−4	A -> G	CAP site	PL11, PL17, PL19, PL24, PL42	4
	+187	C -> A	Box C (DAS)		
**LTR_E**	−4	A -> G	CAP site	PL9, PL18	4
	+5	C -> T	putative GR & PR site		
	+187	C -> A	Box C (DAS)		
**LTR_F**	+161	T -> C	DAS	PL13, PL20, PL30, PL32, PL44	4
**LTR_G**	−10	A -> G	CAP site	PL35	4
	+161	T -> C	DAS		
	+190	T -> C	Box C (DAS)		
**LTR_H**	the consensus sequence-like			PL41	4
**LTR_I**	−65	T -> C	GRE	PL5	4
**LTR_J**	−142	G -> A	NF-kB-like protein	PL4	4
	−11	T -> C	CAP site		
**LTR_K**	−122	T -> A	E box 2	PL29, PL40, PL47	4
**LTR_L**	−133	G -> C	TRE2	PL14, PL15, PL25, PL27, PL28, PL36, PL38, PL39, PL46	7
	+11	C -> T	putative Sp1 & GR site		
	+150	A -> G	Box A (DAS)		
	+160	C -> T	DAS		
**LTR_M**	+11	C -> T	putative Sp1 & GR site	PL10	7
	+150	A -> G	Box A (DAS)		

Abbreviations: NF-κB-like protein, nuclear factor-like protein binding site; TRE2, Tax responsive element 2; GRE, glucocorticoid response element; DAS, downstream activator sequence; putative GR & PR site, putative glucocorticoid response element and progesterone response element site; putative Sp1 & GR site, Specific protein 1 and glucocorticoid response element site.

**Table 4:** Mutations identified in regulatory regions and domains of Tax, miRNA and LTR sequence variants derived from cattle with low PL (WBC < 22 000 per μL and PVL < 100 copy number per 1 000 cells).

Gene/Region	Variant	Mutation	Regulatory Domains/Elements
**Tax**	Tax H	F(178)Y I(246)T, F(249)L	CTL epitope & Leucine-rich domain Multifunctional domain
	Tax I	-	-
	Tax N	R(43)K V(212)I	Zn finger domain adjacent to CTL epitope
	Tax E	-	-
**miRNA**	miRNA_E	G(141)A	B2 pre-miRNA
	miRNA_H	A(420)H	putative B-box
	miRNA_F	G(141)A	B2 pre-miRNA
	miRNA_I	A(420)H A(467)G	putative B-box BLV-mir-B5-5p & putative B-box
	miRNA_J	C(239)T	BLV-mir-B3-5p
**LTR**	LTR_J	G(−142)A T(−11)C	NF-kB-like protein CAP site
	LTR_I	T(−65)C	GRE
	LTR_M	-	-
	LTR_E	A(−4)G	CAP site
	LTR_D	A(−4)G	CAP site

**Table 5:** Mutations identified in regulatory regions and domains of Tax, miRNA and LTR sequence variants derived from cattle with high PL (WBC ≥ 22 000 per μL and PVL ≥ 100 copy number per 1 000 cells).

Gene/Region	Variant	Mutation	Regulatory Domains/Elements
**Tax**	Tax O	-	-
	Tax L	-	-
	Tax B	E(42)K, V(142)A	Zn finger domain, T-cell epitope
	Tax M	A(198)T	CD8+ T-cell epitope
	Tax G	-	-
	Tax C	E(51)K, I(131)V	Zn finger domain, T-cell epitope
	Tax J	Q(117)R	T-cell epitope
**miRNA**	miRNA_K	C(249)G G(181)A	BLV-mir-B3-5p, putative B-box
	miRNA_A	A(310)del *, G(342)A, A(357)G, A(462)G, GA(497–498)AG	BLV-mir-B4-5p, B4 pre-miRNA with putative A-box, BLV-mir-B4-3p, BLV-mir-B5-5p, BLV-mir-B5-3p
	miRNA_B	-	-
	miRNA_D	C(249)G	BLV-mir-B3-5p
	miRNA_C	A(154)G	BLV-mir-B2-3p
**LTR**	LTR F	-	-
	LTR L	G(−133)C, C(+160)T	CRE2, DAS
	LTR A	G(−133)A, C(+183)T	CRE2, DAS
	LTR K	T(−122)A	E box2
	LTR H	-	-
	LTR C	-	-
	LTR B	G(−133)A, A(+150)C, C(+183)T, C(+185)T	CRE2, Box A (DAS), DAS, Box C (DAS)

**-**, lack of characteristic mutation for the variant; *, deletion.

**Table 6:** The relationship between predominant mutations in Tax, miRNA and LTR sequences and the PL stage.

Region	Predominant Residue (Position)		Chi-Square *p*-Value
	Low PL (Group I)	High PL (Group II)	
**Tax**	I(214)	T(214)	0.11416
	T(221)	S(221)	0.34776
	L(233)	P(233)	0.35330
	S(281)	P(281)	0.35331
**miRNA**	del(168)	T(168)	0.13392
	A(441)	G(441)	0.11976
**LTR**	A(+187)	C(+187)	0.00737 *

del, deletion; *, statistically significant finding.

**Table 7:** Description of cattle with persistent lymphocytosis used in this study.

Cattle #	Lymphocyte × 10^3^/µL	WBC × 10^3^/µL	Clinical Stage	Breed	Herd #	Proviral Copy Number per 10^3^ Cells
AL1	8.8	14	AL	B-a-W	I	0.01
AL2	7.6	11	AL	B-a-W	I	224.8
AL3	9.2	14	AL	B-a-W	I	0.7
PL4	11.2	16	PL	B-a-W	I	1.1
PL5	12.8	17	PL	RedStep	II	52.4
PL6	12.2	17	PL	B-a-W	I	0.01
PL7	11.9	17	PL	Ayrshire	III	0.1
PL8	12.4	17.5	PL	RedStep	II	0.3
PL9	14.4	17.5	PL	B-a-W	IV	0.4
PL10	10.6	18	PL	RedStep	II	1.1
PL11	11.7	18	PL	B-a-W	IV	1.3
PL12	9.4	18	PL	B-a-W	IV	1.8
PL13	10.3	18	PL	Ayrshire	III	3.9
PL14	12.2	18	PL	Ayrshire	III	8.4
PL15	12.6	18.3	PL	Ayrshire	III	16.0
PL16	14.3	18.3	PL	B-a-W	IV	23.6
PL17	11.5	18.3	PL	B-a-W	IV	50.7
PL18	14.1	18.5	PL	B-a-W	IV	76.1
PL19	12.0	18.5	PL	B-a-W	IV	90.8
PL20	12.0	19	PL	RedStep	II	96.6
PL21	14.3	19	PL	Ayrshire	III	112.6
PL22	13.1	19	PL	B-a-W	IV	150.8
PL23	13.3	19.5	PL	B-a-W	IV	159.1
PL24	18.3	19.5	PL	B-a-W	IV	160.1
PL25	9.0	19.5	PL	Ayrshire	III	226.7
PL26	13.4	20	PL	B-a-W	IV	271.3
PL27	11.4	20	PL	Ayrshire	III	300.0
PL28	13.6	20	PL	RedStep	II	330.2
PL29	14.3	21	PL	Ayrshire	III	41.6
PL30	12.2	21	PL	Ayrshire	III	51.1
PL31	14.9	21	PL	B-a-W	I	5.3
PL32	18.0	22	PL	RedStep	II	266.2
PL33	15.0	25	PL	B-a-W	IV	235.2
PL34	16.2	24.6	PL	B-a-W	IV	314.6
PL35	19.2	26	PL	RedStep	II	35.2
PL36	16.5	28.5	PL	Ayrshire	III	44.9
PL37	22.3	29	PL	B-a-W	I	312.3
PL38	23.3	31	PL	Ayrshire	III	201.2
PL39	27.2	32	PL	RedStep	II	228.4
PL40	27.5	34	PL	RedStep	II	62.9
PL41	27.2	34	PL	B-a-W	I	359.9
PL42	25.6	35	PL	B-a-W	IV	109.4
PL43	25.6	35	PL	B-a-W	IV	222.4
PL44	21.6	36	PL	Ayrshire	III	182.8
PL45	27.7	36	PL	B-a-W	IV	217.1
PL46	30.0	38	PL	Ayrshire	III	143.6
PL47	34.0	42	PL	RedStep	II	284.4
LE48	44.8	56	LE	B-a-W	IV	175.0

Abbreviations: AL, aleukemic form infection; PL, persistent lymphocytosis cattle; LE, leukemic cattle; B-a-W, Holstein-Friesian breed; RedStep, Red cattle. The cows were 2–4 years old. The PL stage of BLV infection was evaluated according to the lymphocyte count (cells per μL) and the age of each cow according to Bendixen hematological key (<9 000 = normal and >11 000 = lymphocytosis for cows aged 1–2 years; <7 500 = normal and >9 500 = lymphocytosis for cows aged 2–3 years; <6 500 = normal and >8 500 = lymphocytosis for cows aged 3–4 years ).

## Supplementary Materials

The following are available online at https://www.mdpi.com/2076-0817/10/2/246/s1. Figure S1: The histogram shows the distribution of WBCs for 44 samples derived from cattle with persistent lymphocytosis (PL). The bars represents the number of observations (cases) in the analyzed population; Figure S2: Alignment of the translated amino acid sequences of Tax protein from thirty-four Russian BLV strains; Figure S3: Phylogenetic analysis of Tax amino acid sequences; Figure S4: Alignment of miRNA region nucleotide sequences of forty Russian BLV strains; Figure S5: Phylogenetic analysis of miRNA encoding region sequences; Figure S6: Alignment of LTR region nucleotide sequences of forty-one Russian BLV strains; Figure S7: Phylogenetic analysis of LTR sequences; Figure S8: Alignment of Tax (A), miRNA (B) and LTR (C) consensus sequences generated for variants corresponding to the group of cattle with low and high lymphocytosis; Figure S9: Correlation between lymphocyte count and WBC count (r = 0.965, *p* < 0.0000001); Table S1: K-means cluster analysis (A), plot of means for each cluster (B); Table S2: Sequence of primers used to amplify the BLV miRNA encoding region and bovine H3F3A housekeeping gene.
