# Supplementary material for: Analysis of Nucleotide Sequence of Tax, miRNA and LTR of Bovine Leukemia Virus in Cattle with Different Levels of Persistent Lymphocytosis in Russia
Source: Pathogens. 2021 Feb 20;10(2):246. doi: 10.3390/pathogens10020246 (PMC7924208; doi:10.3390/pathogens10020246)
Supplement: Supplementary file 1 [file pathogens-10-00246-s001.zip › pathogens-1091729-supplementary/pathogens-1091729-supplementary figures.docx]

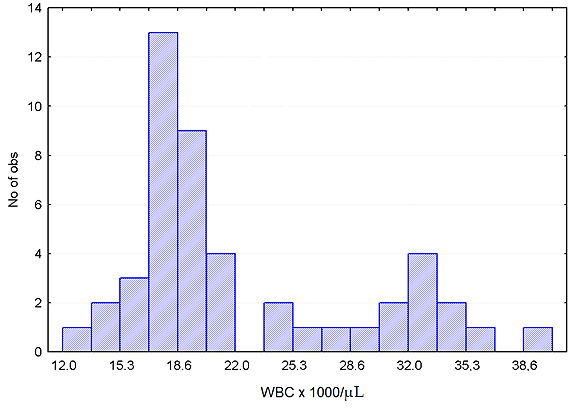


**Figure S1.** The histogram shows the distribution of WBCs for 44 samples derives from cattle with persistent lymphocytosis (PL). The bars represents the number of observations (cases) in the analyzed population.


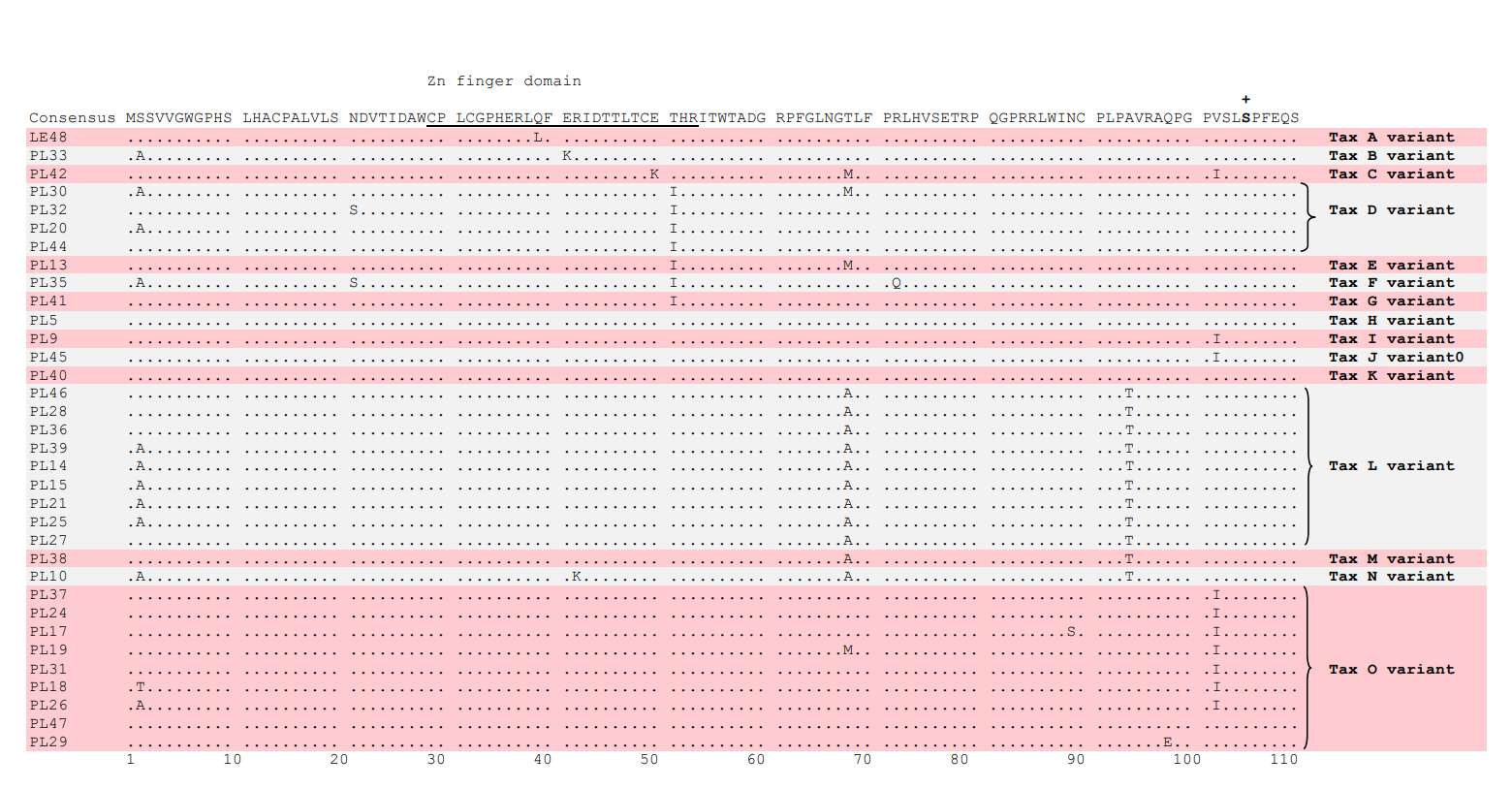


**
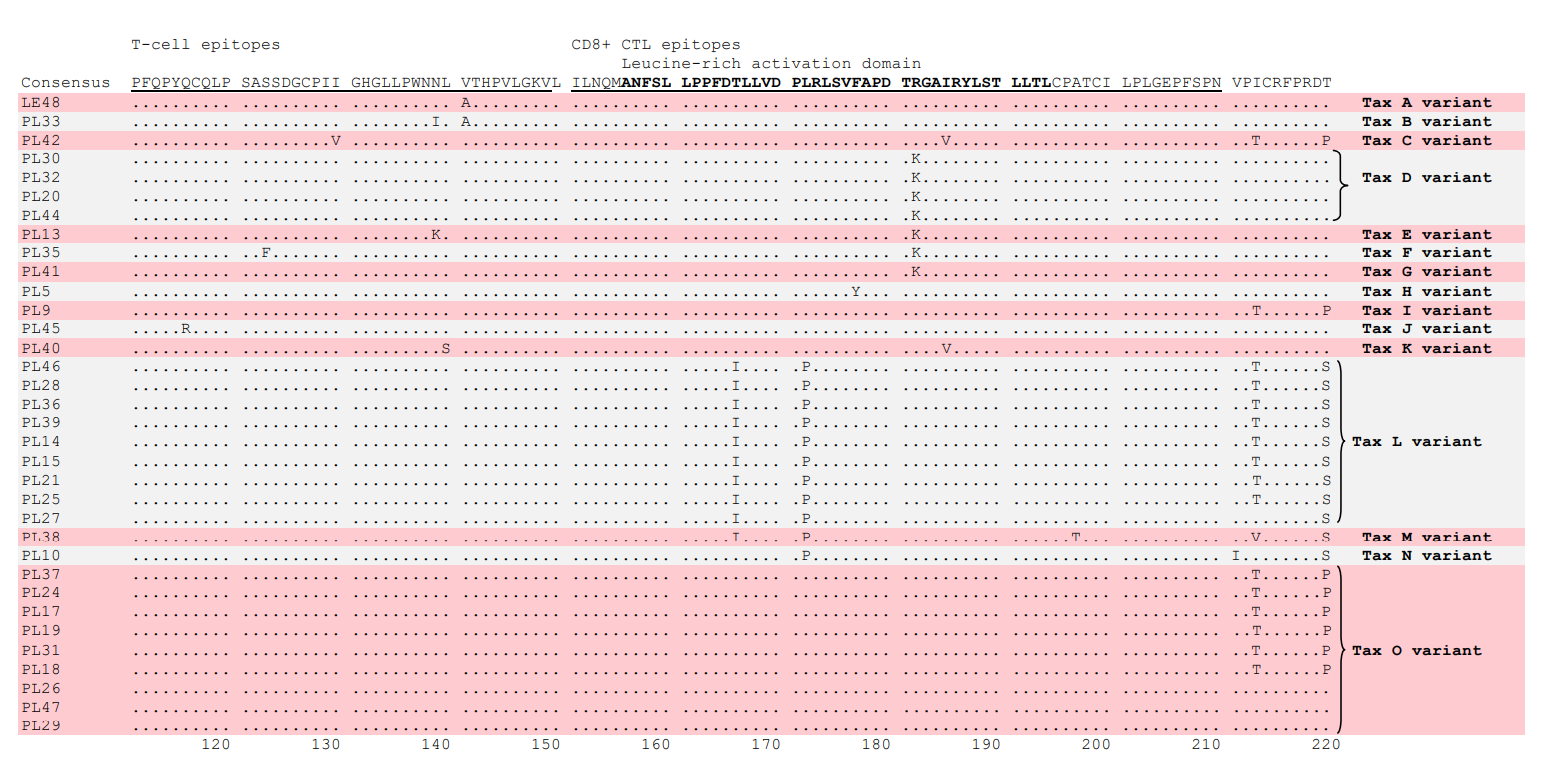

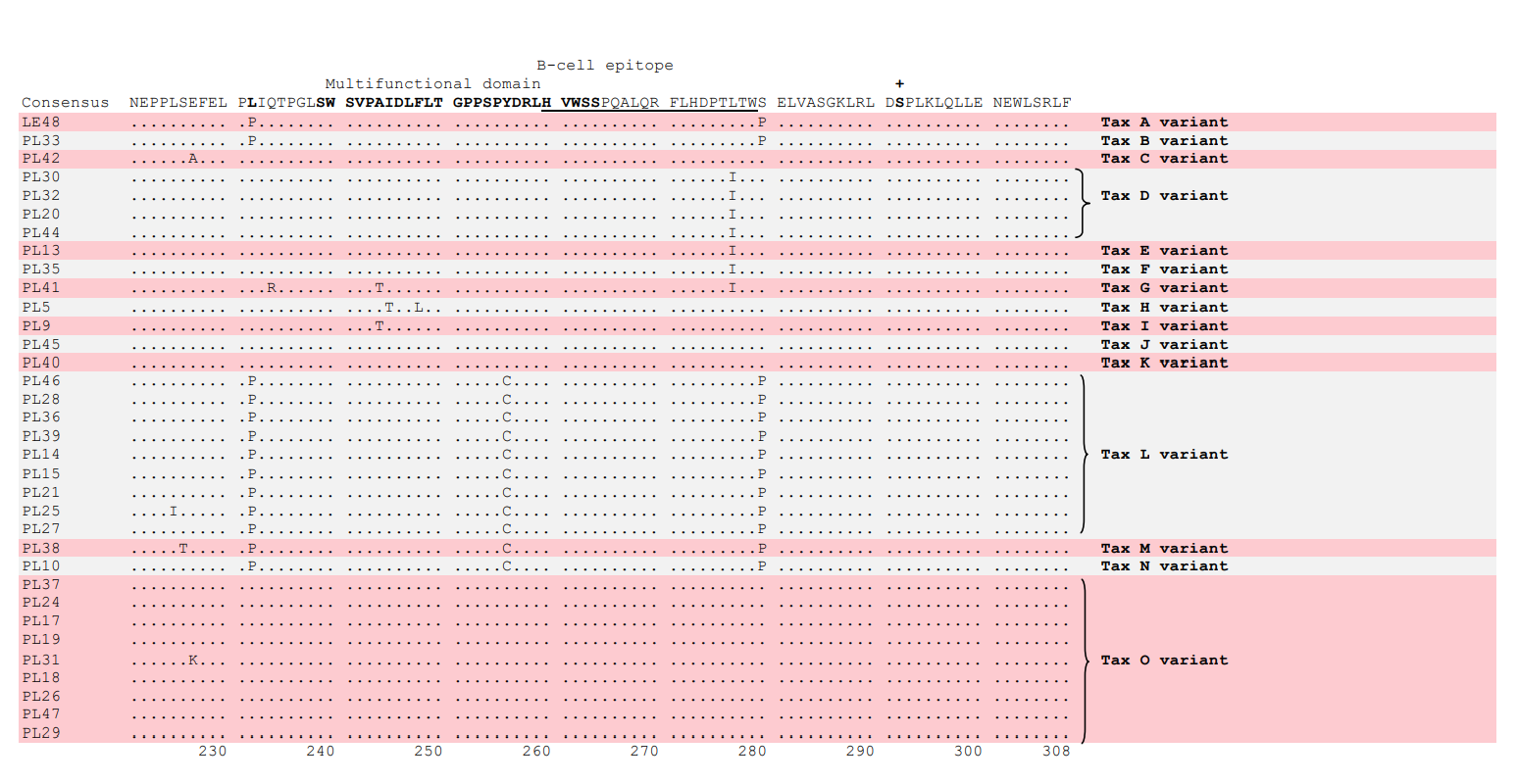
Figure S2.** Alignment of the translated amino acid sequences of Tax protein from thirty-four Russian BLV strains. The points at which translated proviral Tax sequences differ from the consensus are indicated below the consensus sequence. Functionally important regions Zn finger domain, phosphorylation sites, T-cell epitopes, B-cell epitope and CD8+ CTL epitopes are marked with solid black lines. Leucine-rich activation domain and Multifunctional domain are marked in bold in the consensus sequence. Tax variants found in this study are indicated at the right by vertical buckles.


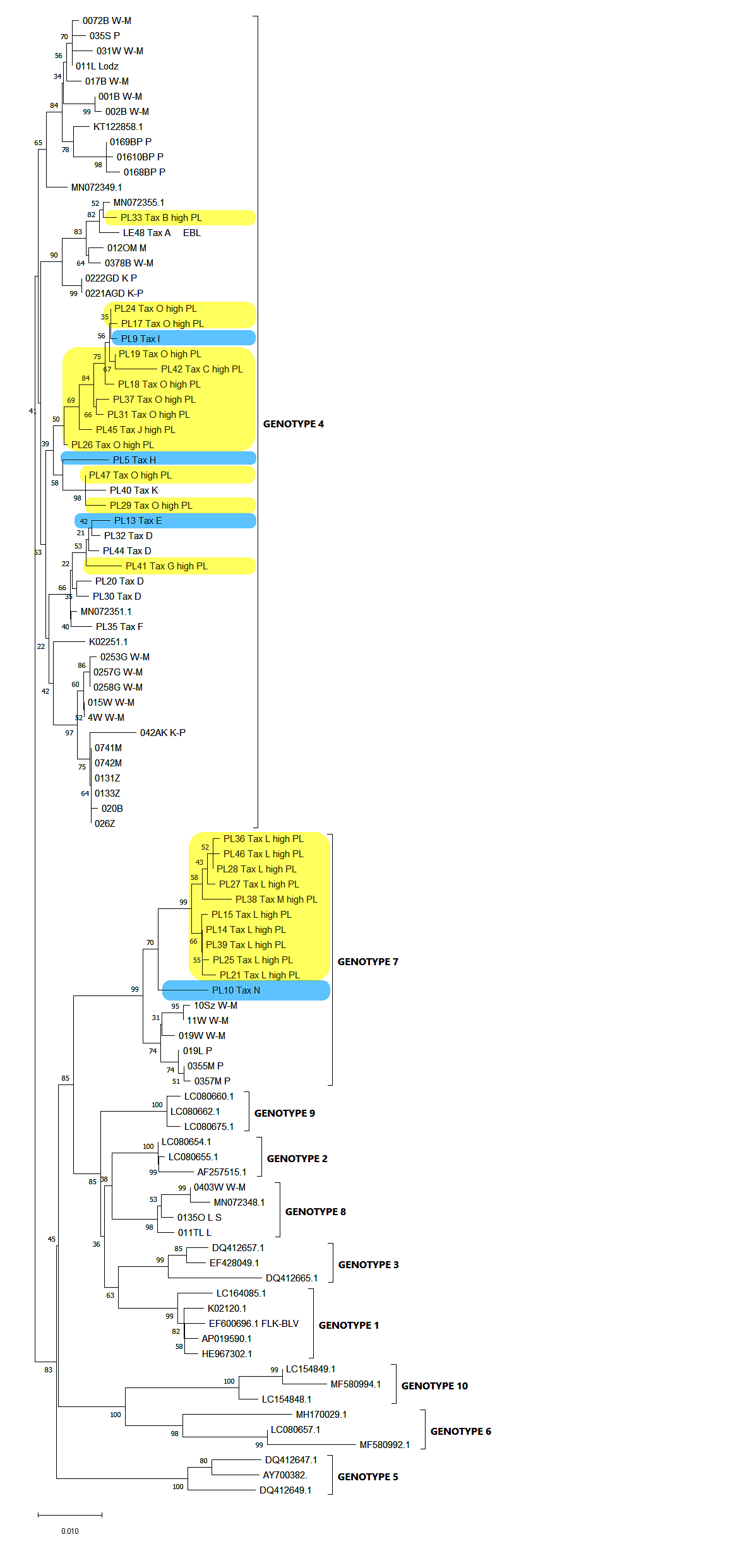


**Figure S3.** Phylogenetic analysis of Tax amino acid sequences. The evolutionary history was inferred using the Neighbor-Joining method. The percentage of replicate trees in which the associated taxa clustered together in the bootstrap test (1000 replicates) are shown next to the branches. The evolutionary distances were computed using the Maximum Composite Likelihood method and are in the units of the number of base substitutions per site. This analysis involved 98 nucleotide sequences. Mean distance within Russian sequences group (*n* = 34) was 0.02299 and within a group consisting of the remaining sequences from other countries (*n* = 64) was 0.03384. Mean distance between these groups was 0.03204. Evolutionary analyses were conducted in MEGA X. Tax variants classified to the low PL group were marked in the blue cloud, the variants assigned to high PL were marked in yellow cloud.


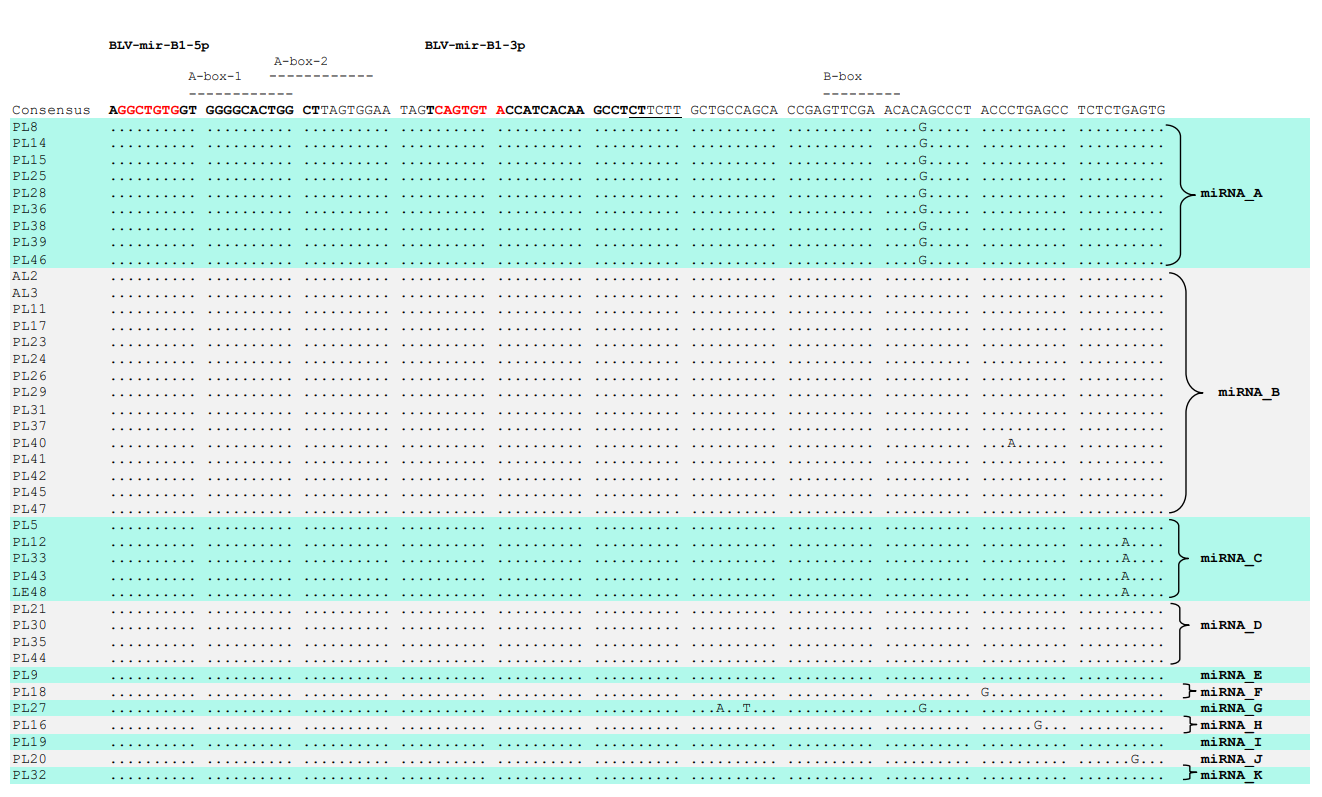

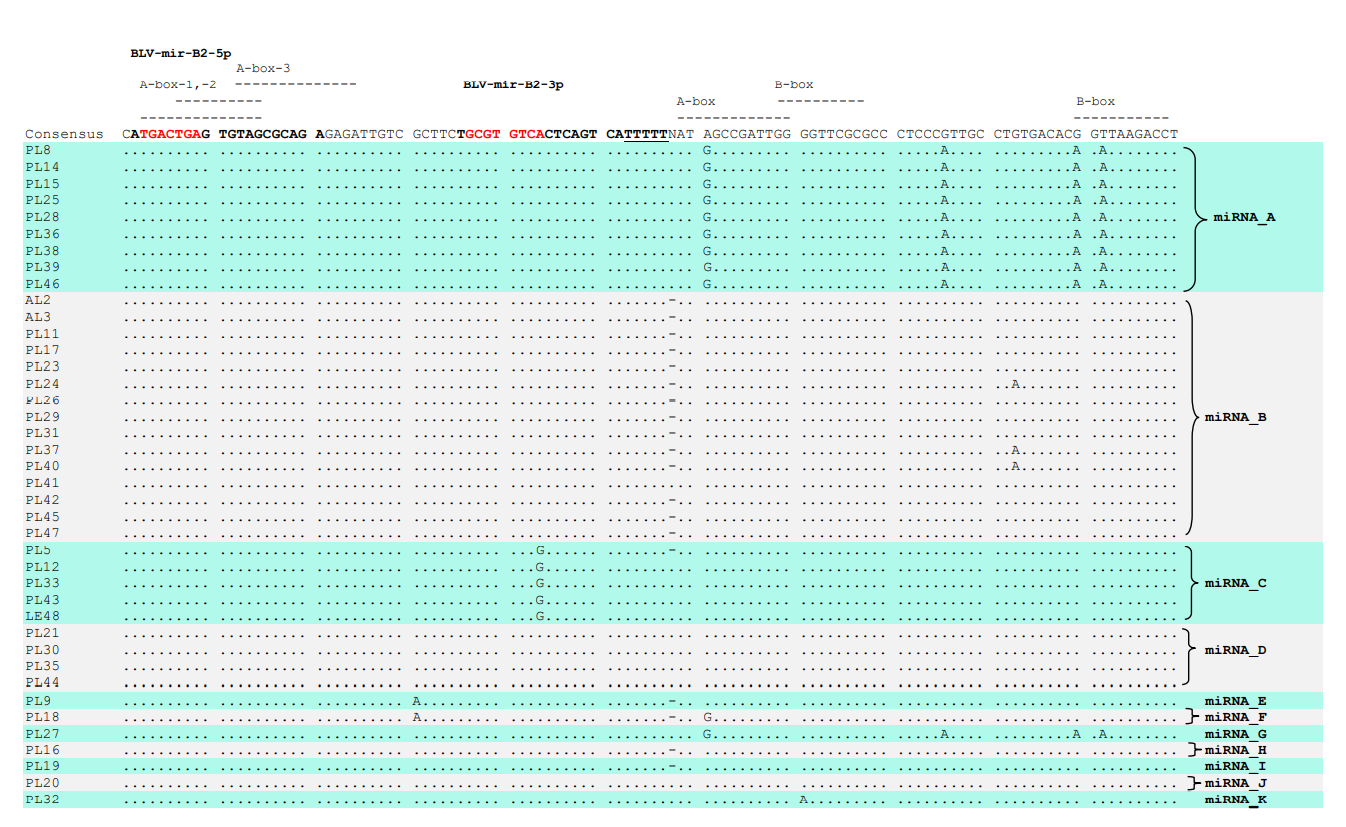

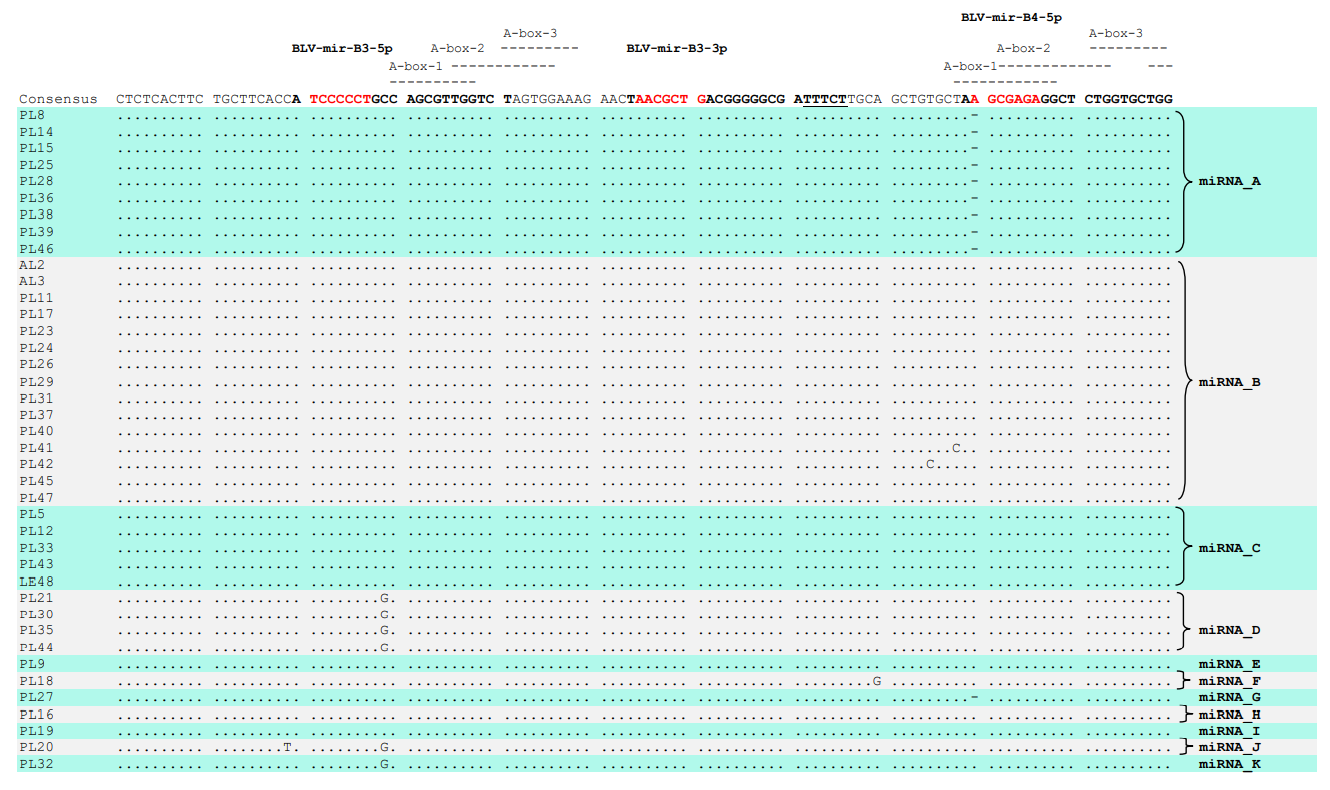

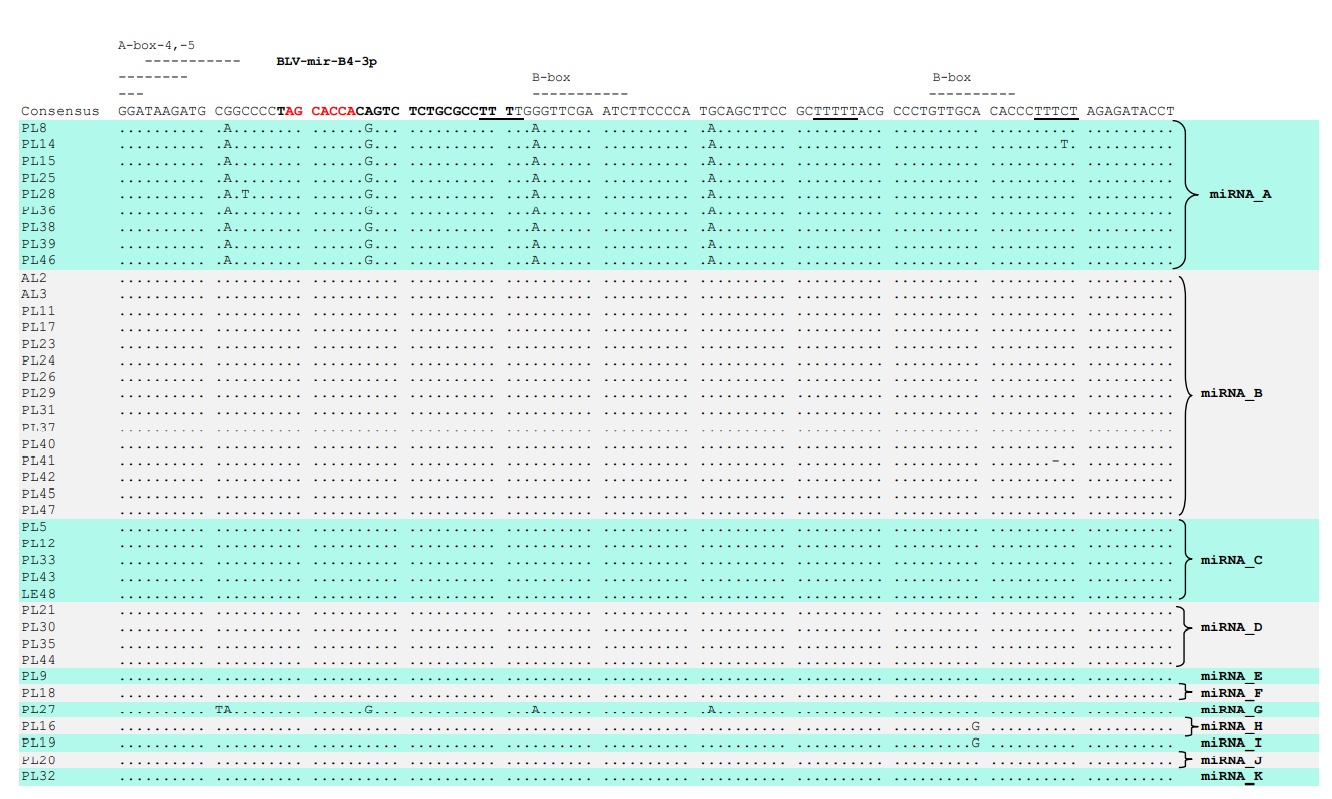

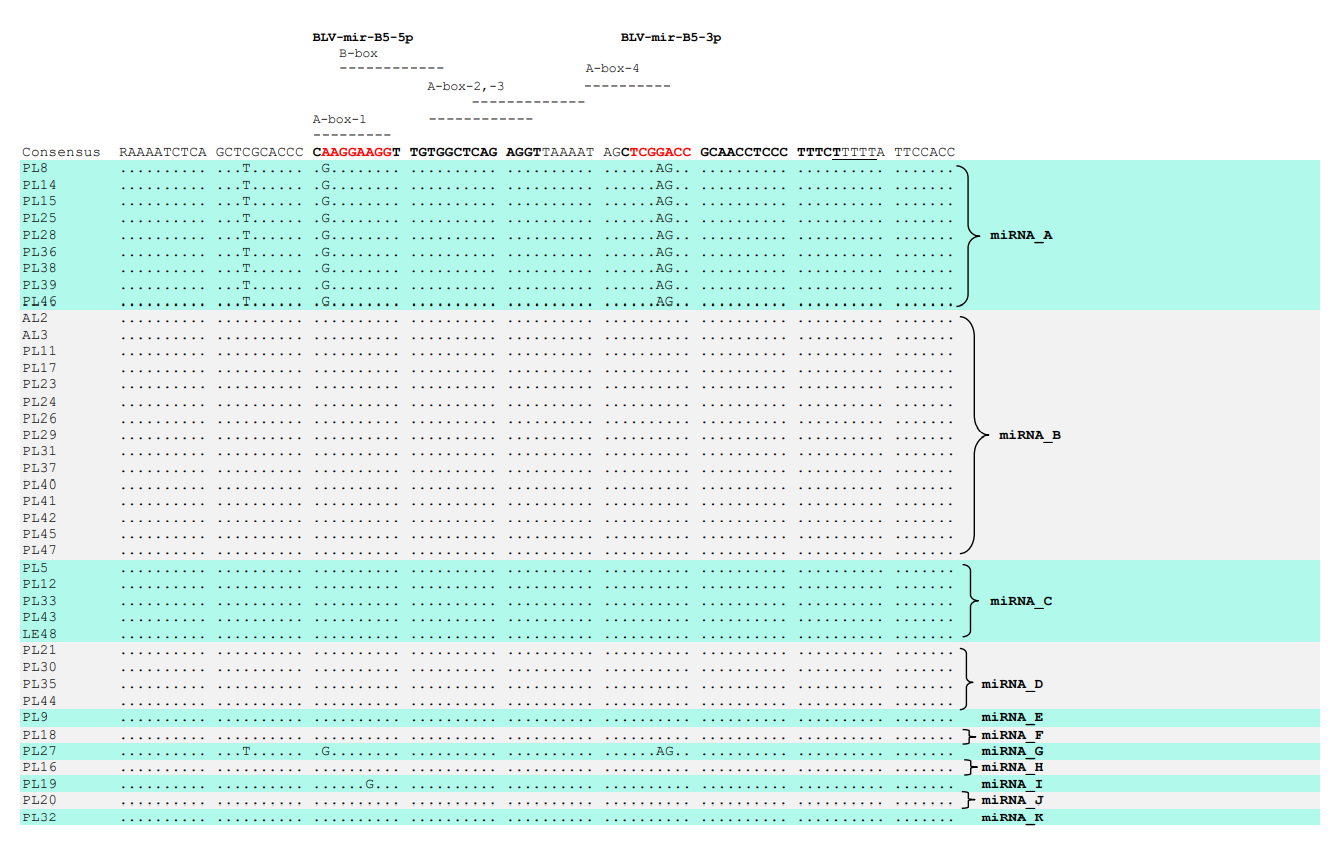


**Figure S4.** Alignment of miRNAs region nucleotide sequences of forty Russian BLV strains. Divergences from the consensus sequence are indicated. Horizontal dashed lines above the nucleotide sequence alignment indicate the putative promoter sequences; putative terminator sequences are marked with solid black lines; miRNA are marked in bold in the consensus sequence; the “seed” regions of miRNA are marked in red in the consensus sequence. The miRNA variants found in this study are indicated at the right by vertical buckles.


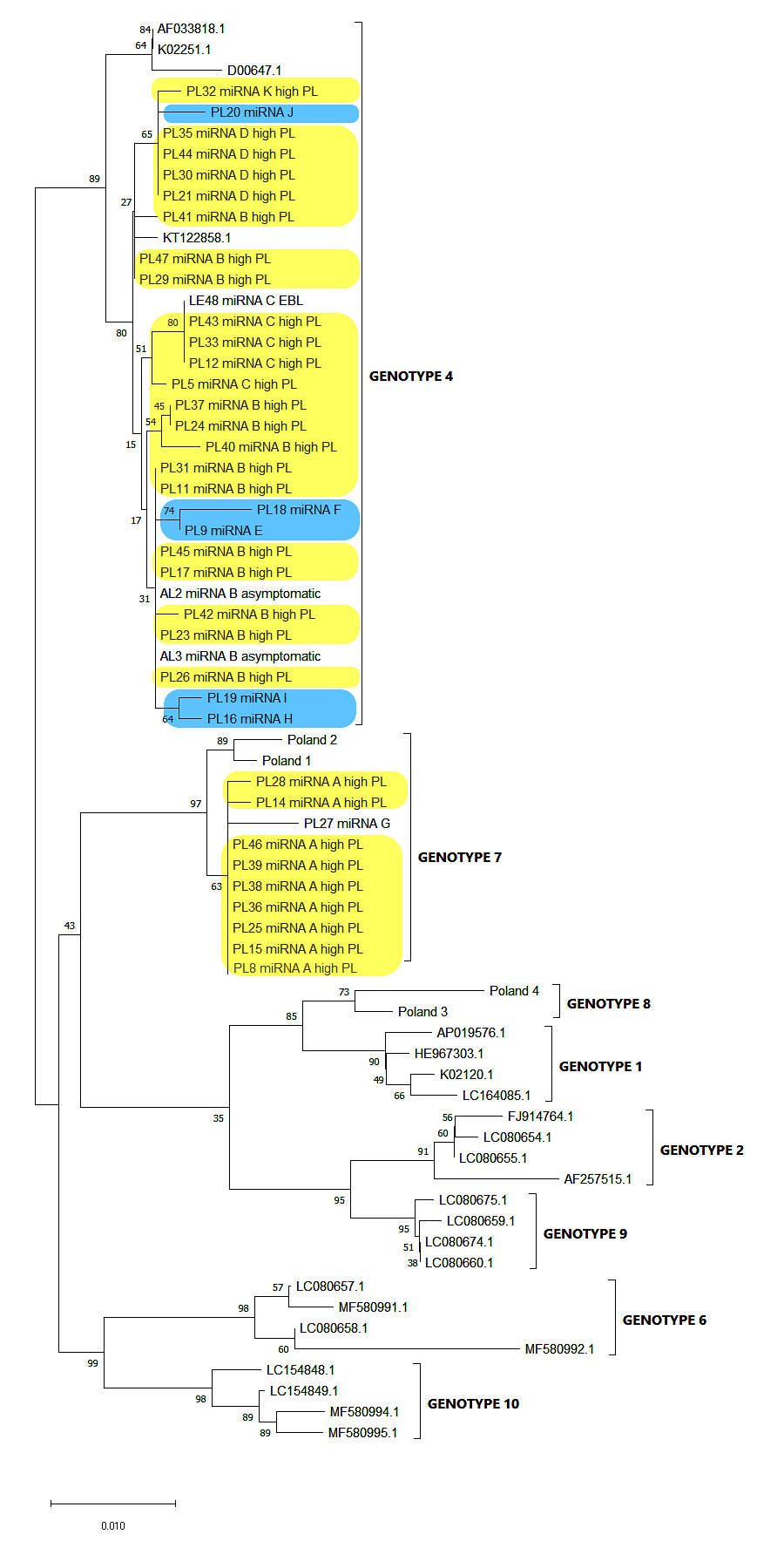


**Figure S5.** Phylogenetic analysis of miRNAs encoding region sequences. The evolutionary history was inferred using the Neighbor-Joining method. The percentage of replicate trees in which the associated taxa clustered together in the bootstrap test (1000 replicates) are shown next to the branches. The evolutionary distances were computed using the Maximum Composite Likelihood method and are in the units of the number of base substitutions per site. This analysis involved 68 nucleotide sequences. Mean distance within Russian sequences group (*n* = 40) was 0.01364 and within a group consisting of the remaining sequences from other countries (*n* = 28) was 0.04005. Mean distance between these groups was 0.03500. Evolutionary analyses were conducted in MEGA X. The miRNA variants classified to the low PL group are marked in the blue cloud, the variants assigned to high PL are marked in yellow cloud. Sequences: Poland 1-Poland 4 (Acc no.: MW470848- MW470851).


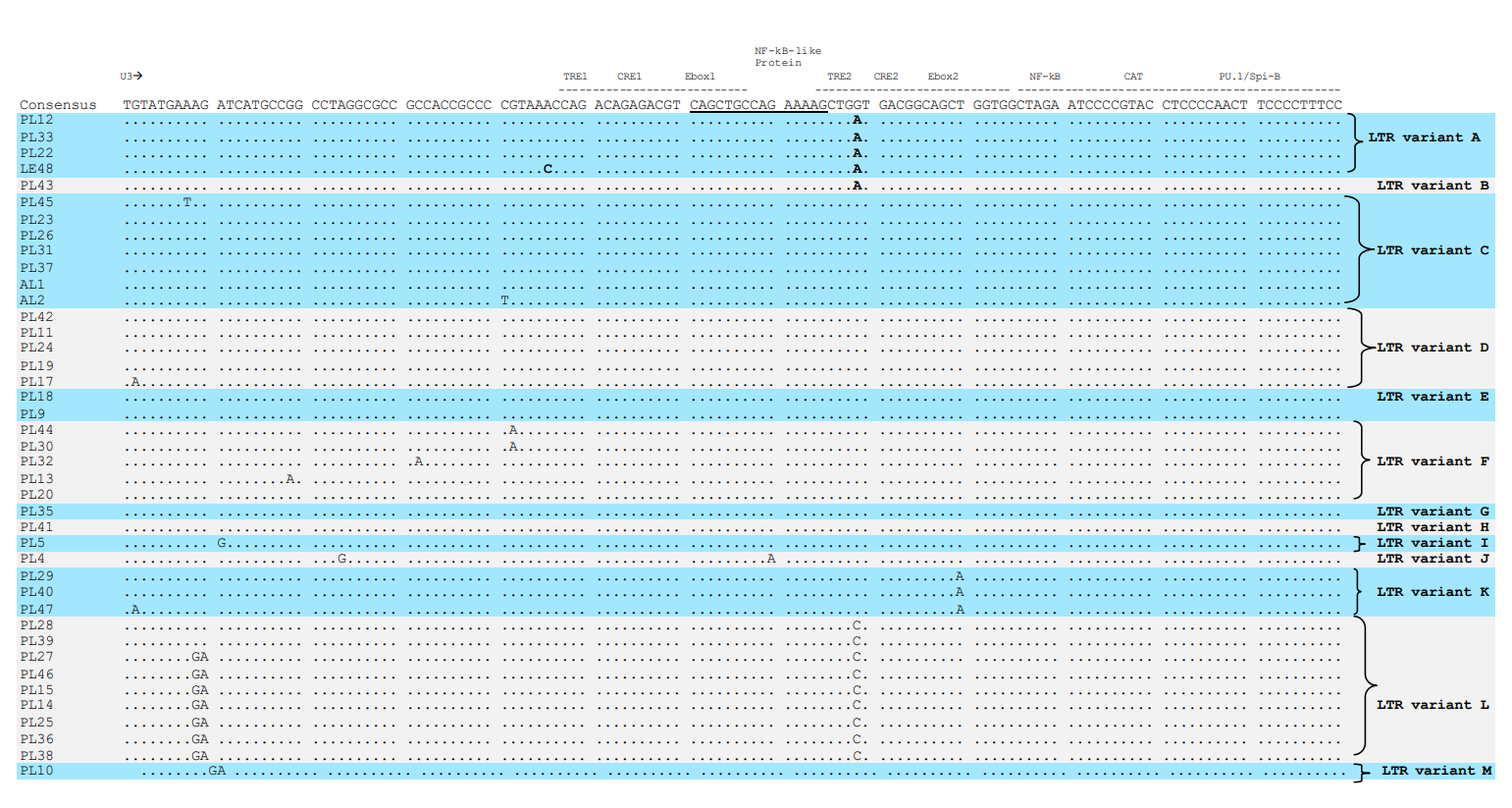

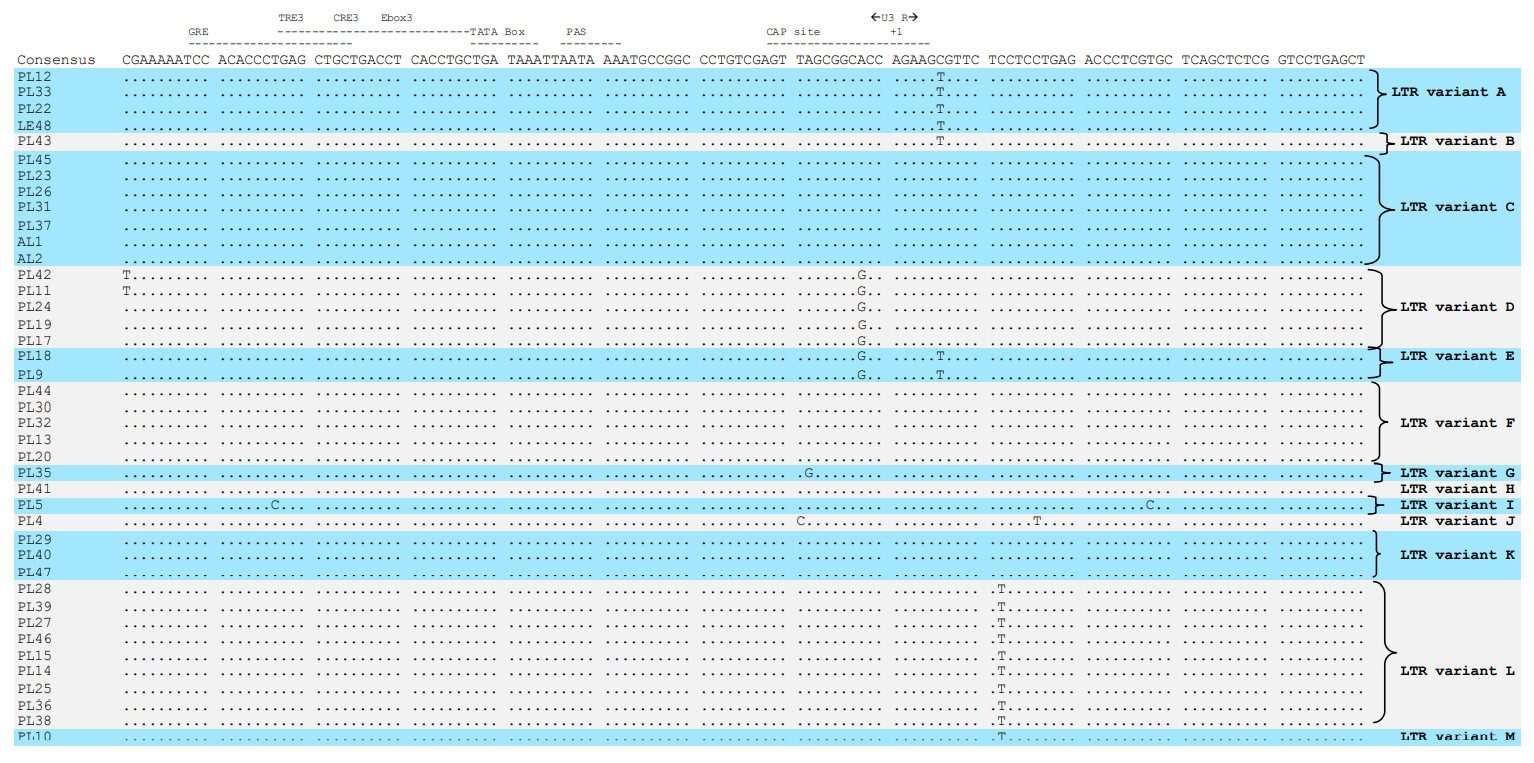

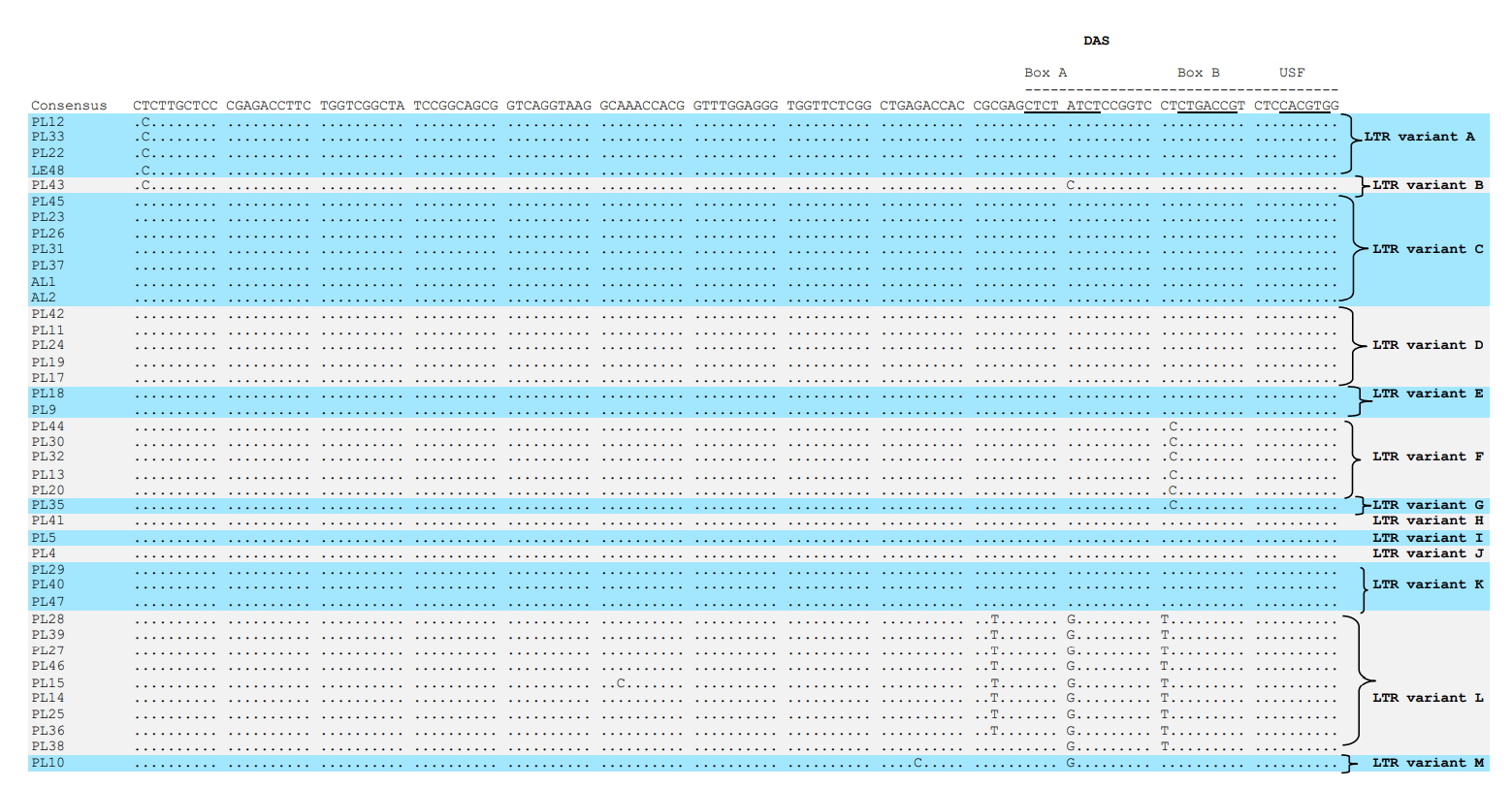

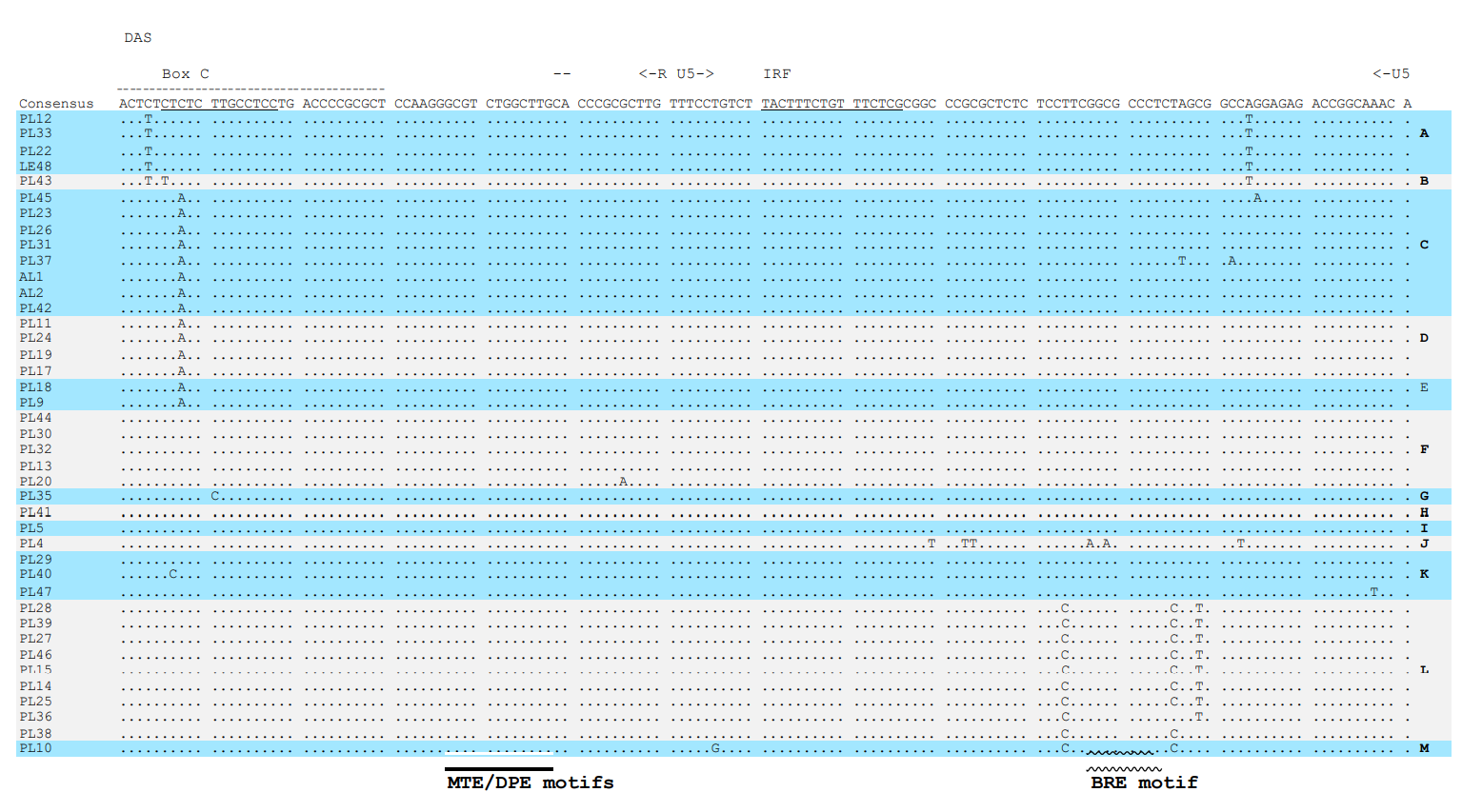


**Figure S6.** Alignment of LTR region nucleotide sequences of forty-one Russian BLV strains. Divergence from the consensus sequence is indicated. Distribution of corresponding regulatory elements along the LTR are indicated in the header. Horizontal dashed lines above the nucleotide sequence alignment indicate the TxRE, Ebox, TATA box, κB, GRE, PU.1/Spi-B, CAT, CAP site and IRF. Solid line below the consensus sequence indicate κB-like site, boxes from A to C within DAS and USF. MTE/DPE and BRE motifs are indicated by black lines below the PL10 sequence. The LTR variants found in this study are indicated at the right by vertical lines.


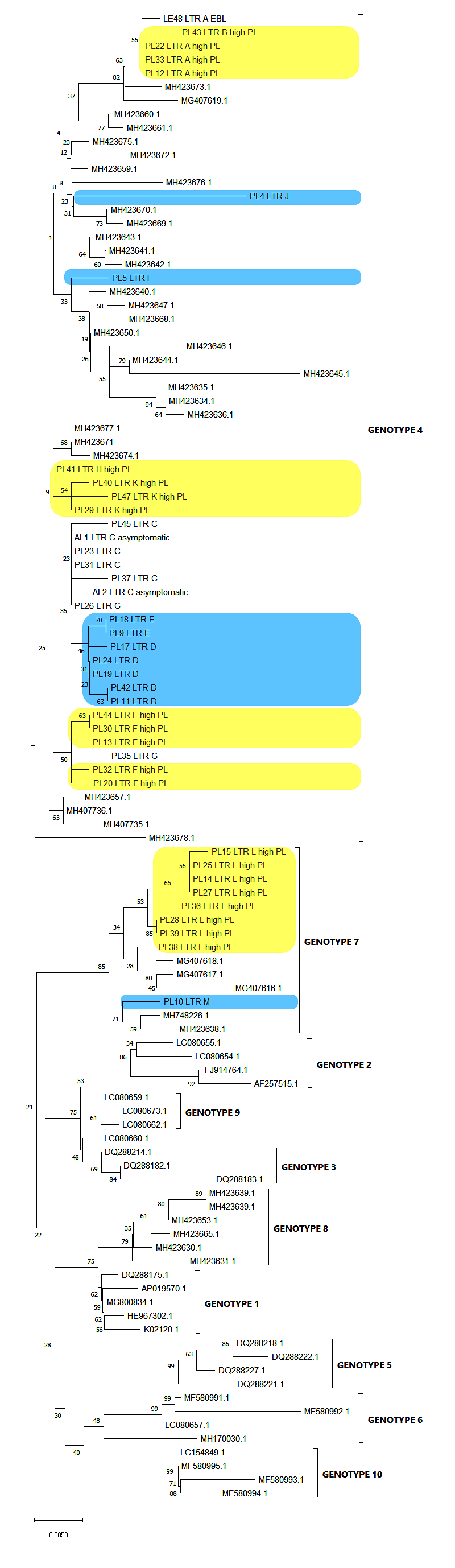


**Figure S7.** Phylogenetic analysis of LTR sequences. The evolutionary history was inferred using the Neighbor-Joining method. The percentage of replicate trees in which the associated taxa clustered together in the bootstrap test (1000 replicates) are shown next to the branches. The evolutionary distances were computed using the Maximum Composite Likelihood method and are in the units of the number of base substitutions per site. This analysis involved 109 nucleotide sequences. Mean distance within Russian sequences group (*n* = 41) was 0.01443 and within a group consisting of the remaining sequences from other countries (*n* = 68) was 0,02318. Mean distance between these groups was 0.02077. Evolutionary analyses were conducted in MEGA X. LTR variants classified to the low PL group are marked in the blue cloud, the variants assigned to high PL are marked in yellow cloud.


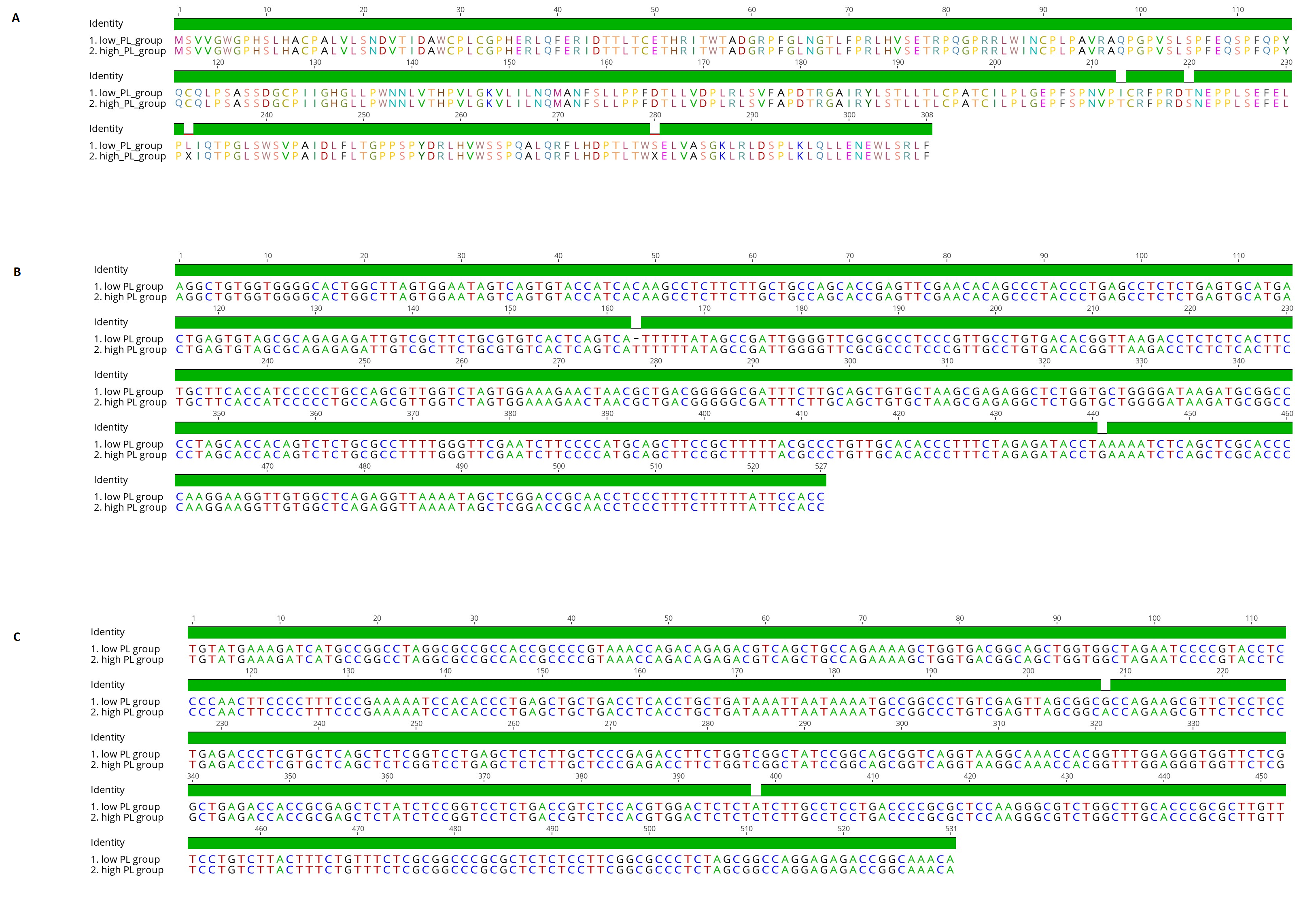


**Figure S8.** Alignment of Tax (A), miRNA (B) and LTR (C) consensus sequences generated for variants corresponding to the group of cattle with low and high lymphocytosis. The green horizontal bar above the alignment indicates the sequence identity. The 233X describe 233P or L, the 281X describe S or P.


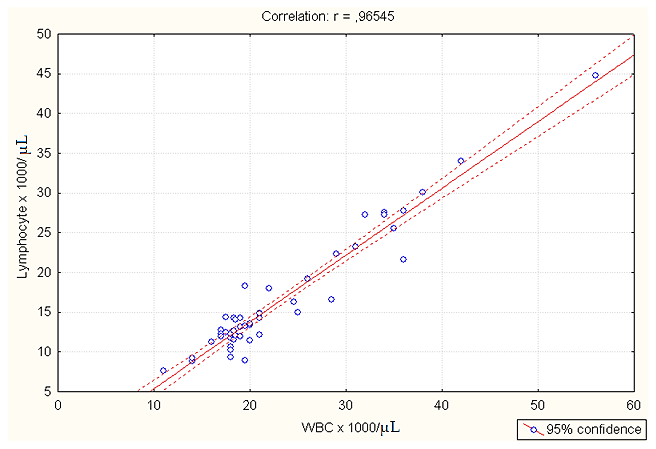


**Figure S9.** Correlation between lymphocyte count and WBC count (r = 0.965, *p* < 0.0000001).
