# Supplementary material for: Analysis of Nucleotide Sequence of Tax, miRNA and LTR of Bovine Leukemia Virus in Cattle with Different Levels of Persistent Lymphocytosis in Russia
Source: Pathogens. 2021 Feb 20;10(2):246. doi: 10.3390/pathogens10020246 (PMC7924208; doi:10.3390/pathogens10020246)
Supplement: Supplementary file 1 [file pathogens-10-00246-s001.zip › pathogens-1091729-supplementary/Table S2.docx]

**Table S2.** Sequence of primers used to amplify the BLV miRNA encoding region and bovine H3F3A housekeeping gene.

| Gene | Name | Location | Sequence |
| --- | --- | --- | --- |
| miRNA | Primers used for first round PCR^ײַ^ | | |
|  | PCR 2118 bp product | | |
|  | P5565 | 5565–5583 | 5’-TGGGTCAACACGTCCTTGT-3’ |
|  | P7660 | 7660–7683 | 5’-GAGGACAGGATGCGTTACTAAGTT-3’ |
|  | Primers used for nested PCR^ײַ^ | | |
|  | PCR 688 bp product | | |
|  | P6308 | 6308–6350 | 5’-GATCTACTCTCACCTCTCCCCC  ACCAAACCCGATTACATCAAC-3’ |
|  | P6952 | 6952–6996 | 5’-ACACTTGGACCTGAGCACCCCC  TAGTAGAGGAAATTTTGAACCTC-3’ |
| H3F3A | Primers and probe used for qPCR^§^ | | |
|  | F_120^Գ^ | 120–139 | 5’-CATGGCTCGTACAAAGCAGA-3’ |
|  | R_236^Գ^ | 236–255 | 5’-ACCAGGCCTGTAACGATGAG-3’ |
|  | Probe | 191–211 | [HEX]-AAGCCGCTCGCAAGAGTGCGC-[BHQ1] |

^ײַ^Numbering of genomic locations was based on the first published complete BLV genome (Sagata et al., 1985; GenBank accession no. K02120). ^§^ Bos taurus H3 histone, family 3A, cDNA clone MGC:157364 (direct submission; GenBank accession no. BC134734. ^Գ^ Bovine H3F3A primers were designed by Meade et al., 2007, *BMC Genomics.*
